# Supplementary material for: Revisiting the impact of synthetic ORF sequences on engineered LINE-1 retrotransposition
Source: PLoS Genet. 2026 Oct 16;22(10):e1012340. doi: 10.1371/journal.pgen.1012340 (PMC13705589; doi:10.1371/journal.pgen.1012340)
Supplement: Supplementary file 1 [file pgen.1012340.s001.pdf]

Supplemental Figure 1

```
                     L1Spa Orf2
L1spa   6742   TCCTGAGTGAGGTAACACAATCACAAAGGAACTCACACAATATGTACTCACTGATAAGTG   6801
               ||||||| ||||| || ||    ||  | |||   ||||| ||||||   ||||| || |
L1SM    6777   TCCTGAGCGAGGTGACCCAGAGCCAGCGCAACAGCCACAACATGTACAGCCTGATCAGCG   6836
               L1SM Orf2
                         SacI   L1spa 3'UTR
L1spa   6802   GATACTAGCGAGCTC-CAAAACCTAGGATACCCACGATATAAGATACAATTTCCTAAACA   6860
               | |||||||       |         ||||||||||||||||||||||||| ||||||||
L1SM    6837   GCTACTAGCCTAAAACCGGT------GATACCCACGATATAAGATACAATTGCCTAAACA   6890
                             AgeI        L1SM 3'UTR

L1spa   6861   CATGAAACTCAAGAAAAATGAAGACTGAAGTGTGGACACTATGCCCCTCCTTAGAAGTGG   6920
               ||||||||||||||||||||||||||||||||||||||||||||||||||||||||||||
L1SM    6891   CATGAAACTCAAGAAAAATGAAGACTGAAGTGTGGACACTATGCCCCTCCTTAGAAGTGG   6950

L1spa   6921   GAACAAAACACCCATGGAAGGAGTTACAGAAACAAAGTTTGGAGCTGAGATGAAAGGAGG   6980
               |||||||||| |||| ||| ||||||||||||||||||||||||||||||||||||||| 
L1SM    6951   GAACAAAACAGCCATAGAAAGAGTTACAGAAACAAAGTTTGGAGCTGAGATGAAAGGAGA   7010

L1spa   6981   GACCATGTAGAGACTGCCATATCCAGGGATCCACCCCATAATCAGCATCCAAACGC--TG   7038
               ||||||||||| |||||||||||||||| |||||||||||||||||||||||  ||  ||
L1SM    7011   GACCATGTAGAAACTGCCATATCCAGGGTTCCACCCCATAATCAGCATCCAA--GCTTTG   7068

L1spa   7039   ACACCATTGCATATACTAGCAAGATTTTATCGAAAGGACCCAGATGTAGCTGTCTCTTGT   7098
               ||||||||||||||||||| ||||||||||||||||||||||||||||||||||||||||
L1SM    7069   ACACCATTGCATATACTAGGAAGATTTTATCGAAAGGACCCAGATGTAGCTGTCTCTTGT   7128

L1spa   7099   GAGACTATGCCGGGGCCTAGCAAACACAGAAGTGGATGCTCACAGTCAGCTAATGGATGG   7158
               ||||||||||||||||||||||| |||||||||||||||||||||||||||| |||||||
L1SM    7129   GAGACTATGCCGGGGCCTAGCAA-CACAGAAGTGGATGCTCACAGTCAGCTA-TGGATGG   7186

L1spa   7159   ATCACAGGGCTCCCAATGGAGGAGCTAGAGAAAGTACCCAAGGAGCTAAAGGGATCTGCA   7218
               |||||||||||  |||||||||||||||||||||||||||||||||||||||||||||||
L1SM    7187   ATCACAGGGCTTTCAATGGAGGAGCTAGAGAAAGTACCCAAGGAGCTAAAGGGATCTGCA   7246

L1spa   7219   ACCCTATAGGTGGAACAACATTATGAAC-TAACCAGTACCCCTGAGCTCTTGACTCTAGC   7277
               || |||||||||||||||||||||||   |||||||||||| |||||||||| | |||||
L1SM    7247   ACTCTATAGGTGGAACAACATTATGA-GTTAACCAGTACCCTTGAGCTCTTGTCGCTAGC   7305

L1spa   7278   TGCATATGTATCAAAAGATGGCCTAGTCGGCCATCACTGGAAAGAGAGGCCCATTGGACA   7337
               ||||||||||
L1SM    7306   TGCATATGTA--------------------------------------------------   7315

L1spa   7338   CGCAGACTTTGTGTGCCCCGGTACAGGGGAACGCCAGGGCCAAAGGGGGGGGAGTGGGTG   7397

L1SM    7316   ------------------------------------------------------------   7315

                                                               PacI
L1spa   7398   GGTAGGGGAGTGGGGGTAGGTGGGTAAGGGGGACTTTTGGTATAGCATTAATTAACTCGA   7457

L1SM    7316   ------------------------------------------------------------   7315

                                       HSV Tk pA (antisense)
L1spa   7458   TACATATGTAACTAACCCGATCCGAACAAACGACCCAACACCCGTGCGTTTTATTCTGTC   7517
                         ||||||||||||||||||||||||||||||||||||||||||||||||||
L1SM    7316   ----------ACTAACCCGATCCGAACAAACGACCCAACACCCGTGCGTTTTATTCTGTC   7365

                                  NEO (antisense)
L1spa   7518   TTTTTATTGCCGATCCCCTCAGAAGAACTCGTCAAGAAGGCGATAGAAGGCGATGCGCTG   7577
               ||||||||||||||||||||||||||||||||||||||||||||||||||||||||||||
L1SM    7366   TTTTTATTGCCGATCCCCTCAGAAGAACTCGTCAAGAAGGCGATAGAAGGCGATGCGCTG   7425
```
