# Supplementary material for: Revisiting the impact of synthetic ORF sequences on engineered LINE-1 retrotransposition
Source: PLoS Genet. 2026 Oct 16;22(10):e1012340. doi: 10.1371/journal.pgen.1012340 (PMC13705589; doi:10.1371/journal.pgen.1012340)

Supplemental Figure 3

A.

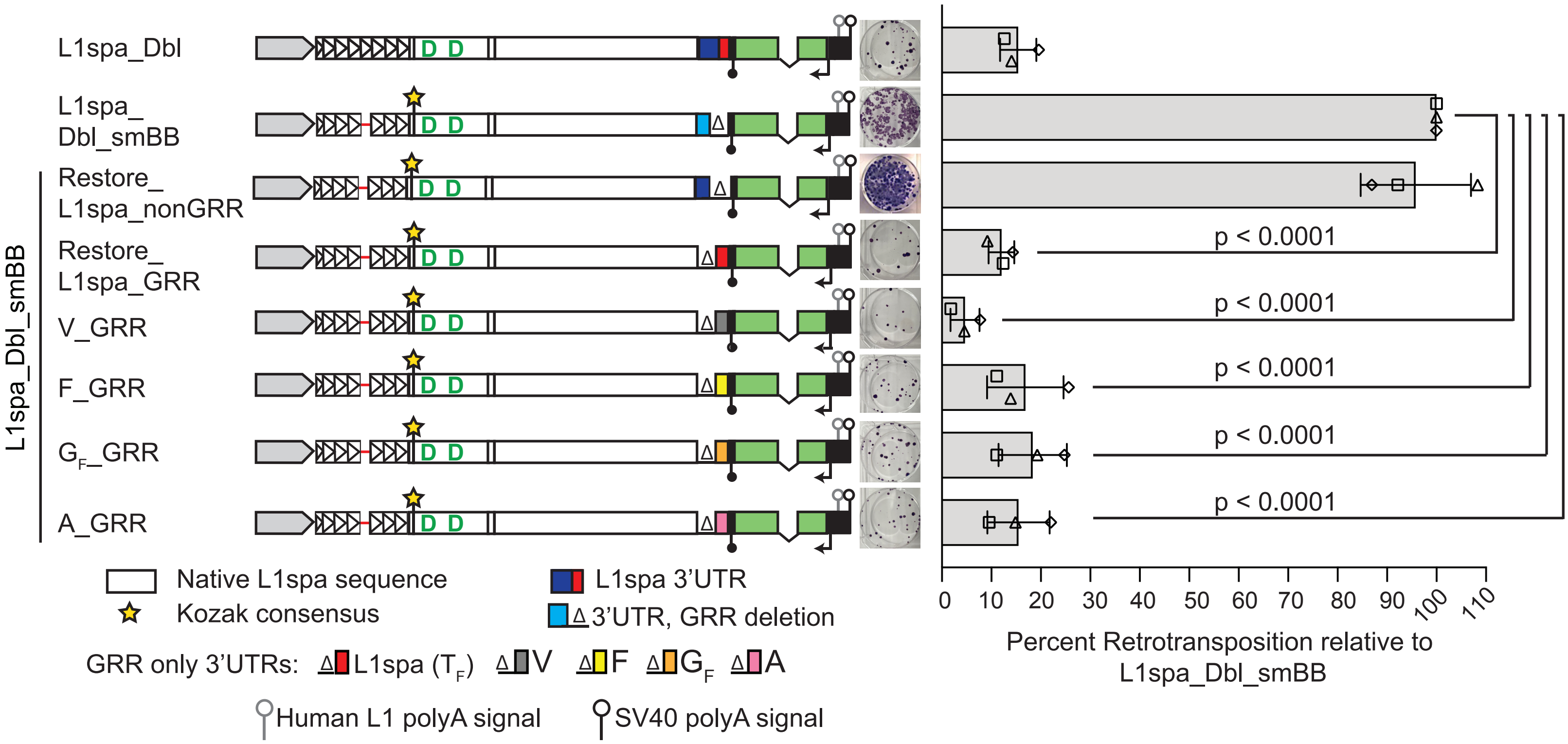

B.

L1spa GRR nucleotide sequence

5′-TCAAAAGATGGCCTAGTCGGCCATCACTGGAAAGAGAGGCCCATTGGACACGCAGA

CTTTGTGTGCCCCGGTACA**GGGG**(G1)AACGCCA**GGG**(G2)CCAAA**GGGGGGGG**(G3)AGT**GGG**(G4)T**GGG**(G5)

TA**GGGG**(G6)AGT**GGGGG**(G7)TAGGT**GGG**(G8)TAA**GGGGG**(G9)ACTTTTGGTATAGCATT-3′

C.

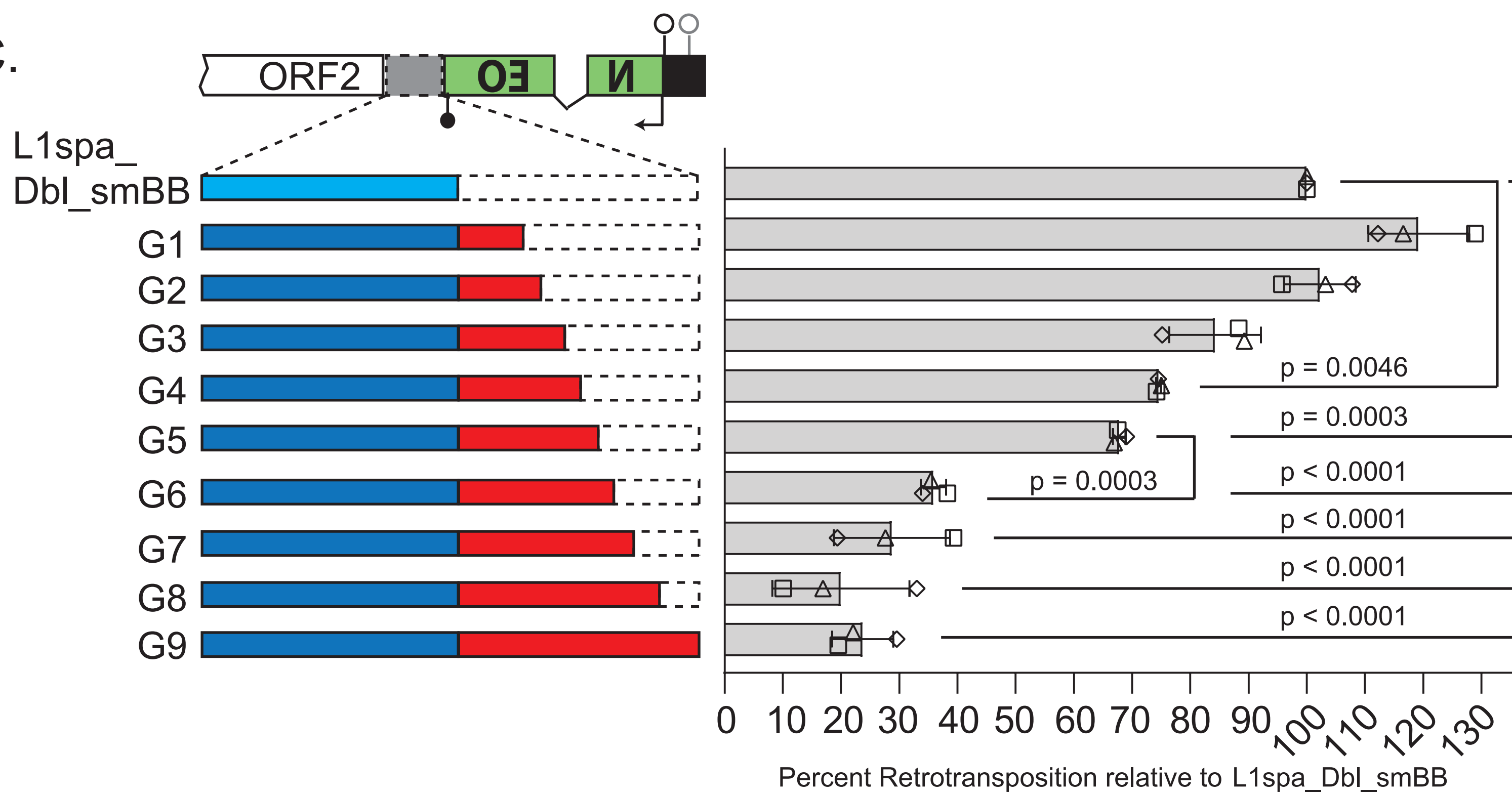

Supplement: Supplementary file 3 [file pgen.1012340.s003.pdf]
