# Supplementary material for: Revisiting the impact of synthetic ORF sequences on engineered LINE-1 retrotransposition
Source: PLoS Genet. 2026 Oct 16;22(10):e1012340. doi: 10.1371/journal.pgen.1012340 (PMC13705589; doi:10.1371/journal.pgen.1012340)

Supplemental Figure 5A

Insertion A

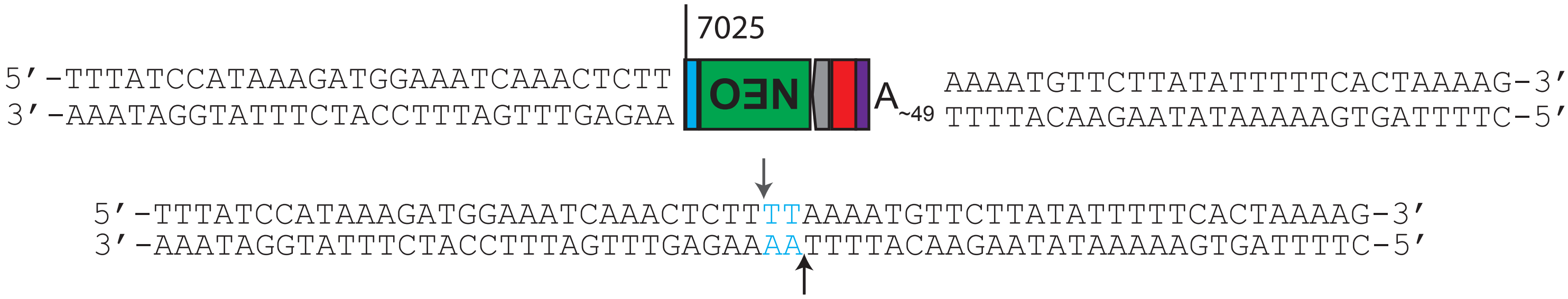

Target-site deletion: Chr3:130997869-130997870

EN cleavage: 5'-TTTTT/AA-3'

Insertion B

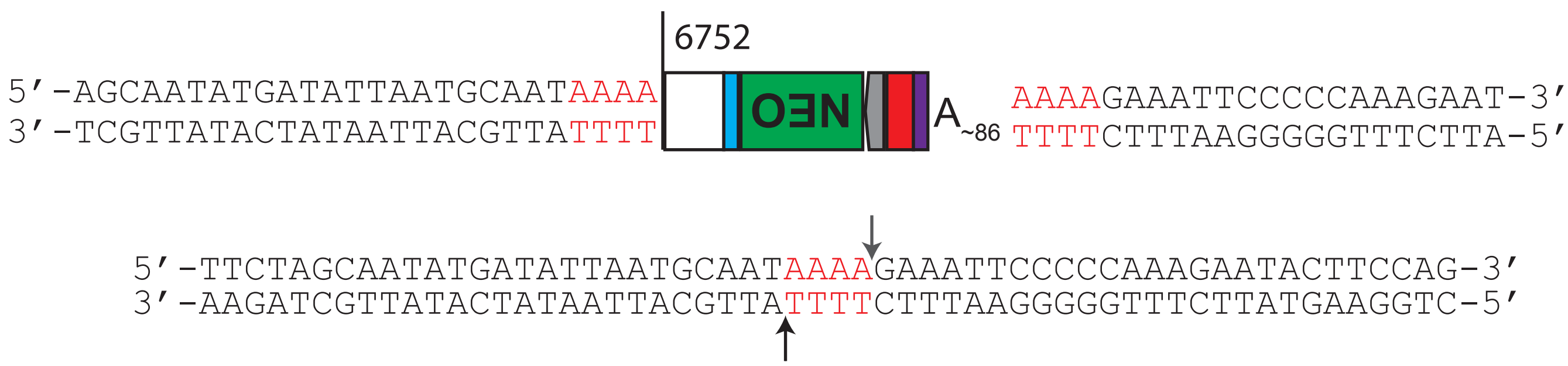

TSD: Chr3:148918423-148918426

EN cleavage: 5'-CTTTT/AT-3'

Insertion C

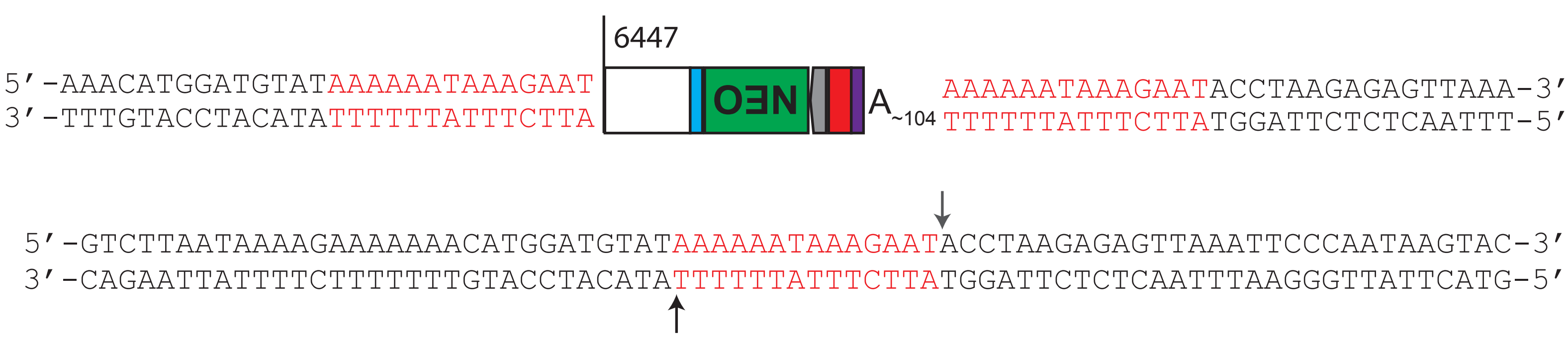

TSD: Chr5: 21539952-21539965

EN cleavage: 5'-TTTTT/AT-3'

Supplemental Figure 5B

Insertion D

```
5′-ATAATTTTTGTGTCATGGTTTATGTTTTT
3′-TATTAAAAACACAGTACCAAATACAAAAA

AAAAAAACTTACCTTTTAACATGCTAAAAAAG
TTTTTTTGAATGGAAAATTGTACGATTTTTTC

ACAGTACTTCTTGAATGGTAGAATAAAGACCA
TGTCATGAAGAACTTACCATCTTATTTCTGGT

TCAAAAATCTGCTCATCCATAAAAGCAATGAG
AGTTTTTAGACGAGTAGGTATTTTCGTTACTC

ATCAGTGGCAAAAATTGTCAAAATTAACTTTT
TAGTCACCGTTTTTAACAGTTTTAATTGAAAA

TCAGAACTCTGGAAATTAACTAAAAGATTGC
AGTCTTGAGACCTTTAATTGATTTTCTAACG
```

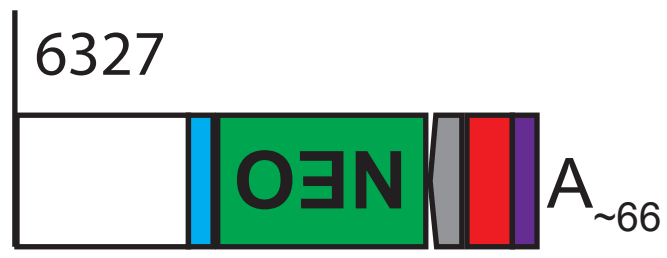


```
AAAAAAACTTACCTTTTAACATGCTAAAAAAG
TTTTTTTGAATGGAAAATTGTACGATTTTTTC

ACAGTACTTCTTGAATGGTAGAATAAAGACCA
TGTCATGAAGAACTTACCATCTTATTTCTGGT

TCAAAAATCTGCTCATCCATAAAAGCAATGAG
AGTTTTTAGACGAGTAGGTATTTTCGTTACTC

ATCAGTGGCAAAAATTGTCAAAATTAACTTTT
TAGTCACCGTTTTTAACAGTTTTAATTGAAAA

TCAGAACTCTGGAAATTAACTAAAAGATTGC
AGTCTTGAGACCTTTAATTGATTTTCTAACG

AAAATGTCAAGGACTGTTTAGTCAGGAAAA-3′
TTTTACAGTTCCTGACAAATCAGTCCTTTT-5′
```

TSD: Chr21: 29204205-29204047

EN cleavage: 5′-TTTTT/AA-3′

```
5′-TGTCATGGTTTATGTTTTTTAAAAAAACTTACCTTTTAACATGCTAAAAAAGACAGTACTTCTTGAATGGTAGAATAAAG
3′-ACAGTACCAAATACAAAAAATTTTTTTGAATGGAAAATTGTACGATTTTTTCTGTCATGAAGAACTTACCATCTTATTTC
                      ↑

ACCATCAAAAATCTGCTCATCCATAAAAGCAATGAGATCAGTGGCAAAAATTGTCAAAATTAACTTTTTCAGAACTCTGG
TGGTAGTTTTTAGACGAGTAGGTATTTTCGTTACTCTAGTCACCGTTTTTAACAGTTTTAATTGAAAAAGTCTTGAGACC

                   ↓
AAATTAACTAAAAGATTGCAAAATGTCAAGGACTGTTT-3′
TTTAATTGATTTTCTAACGTTTTACAGTTCCTGACAAA-5′
```

Insertion E

```
5′-TTTATATAAACAATATATAAACAA
3′-AAATATATTTGTTATATATTTGTT
```

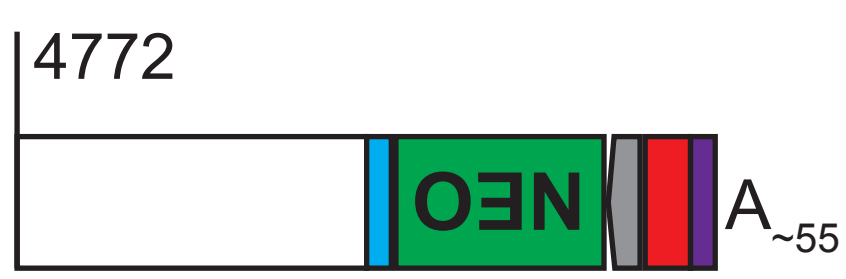


```
ACCTCAGTTAGAATATTTAATTAA-3′
TGGAGTCAATCTTATAAATTAATT-5′
```

```
                           ↓
5′-TTTATATAAACAATATATAAACAACCTCAGTTAGAATATTTAATTAA-3′
3′-AAATATATTTGTTATATATTTGTTGGAGTCAATCTTATAAATTAATT-5′
                           ↑
```

TSD: chr7: 153197463

EN cleavage: 5′-GAGGT/TG-3′

Supplemental Figure 5C

Insertion F

5′-AATTCTTTGGAAAAAAGACAAAGTTGAAATAATGTCAGAGAGAATACATCAGGAAAAAGGTTGAAAATGTAAGTTCTA
3′-TTAAGAAACCTTTTTTCTGTTTCAACTTTATTACAGTCTCTCTTATGTAGTCCTTTTTCCAACTTTTACATTCAAGAT

TTAAACACAACATACTTAGAGGGGGAGAAAATCACAGAAGAGCCAGTTATGGCAGCTGAAAGAGGGGCAATGCTAAAGAC
AATTTGTGTTGTATGAATCTCCCCCTCTTTTAGTGTCTTCTCGGTCAATACCGTCGACTTTCTCCCCGTTACGATTTCTG

TCACATTTTTAATTTAAAAATCAGTGTTCCCTGGGCAGCAAGAAATTTTTGCCTATACTATATACAAAAAATTCATTCAA
AGTGTAAAAATTAAATTTTTAGTCACAAGGGACCCGTCGTTCTTTAAAAACGGATATGATATATGTTTTTTAAGTAAGTT

AGAGAATAAAAGACCTAAACCTGAGAGCTAAAACTCTAAAACCTTTAGAAGAAAACATAAGGGAAATGCTTCATGACATT
TCTCTTATTTTCTGGATTTGGACTCTCGATTTTGAGATTTTGGAAATCTTCTTTTGTATTCCCTTTACGAAGTACTGTAA

GAACTTTACAGTGGTGTCTTGGATATGATACCAAAAGCATGGACAATGAAAGAAAAAAAAACACATAGATTTCATCGAAG
CTTGAAATGTCACCACAGAACCTATACTATGGTTTTCGTACCTGTTACTTTCTTTTTTTTTGTGTATCTAAAGTAGCTTC

TAAAAACTTCTGTGCATTAAGTAACATTC
ATTTTTGAAGACACGTAATTCATTGTAAG

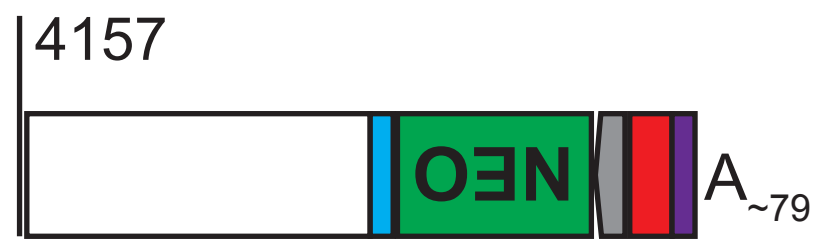

GAAAATGTAAGTTCTATTAAACACAACAT
CTTTTACATTCAAGATAATTTGTGTTGTA

ACTTAGAGGGGGAGAAAATCACAGAAGAGCCAGTTATGGCAGCTGAAAGAGGGGCAATGCTAAAGACTCACATTTTTAAT
TGAATCTCCCCCTCTTTTAGTGTCTTCTCGGTCAATACCGTCGACTTTCTCCCCGTTACGATTTCTGAGTGTAAAAATTA

TTAAAAATCAGTGTTCCCTGGGCAGCAAGAAATTTTTGCCTATACTATATACAAAAAATTCATTCAAAGAGAATAAAAGA
AATTTTTAGTCACAAGGGACCCGTCGTTCTTTAAAAACGGATATGATATATGTTTTTTAAGTAAGTTTCTCTTATTTTCT

CCTAAACCTGAGAGCTAAAACTCTAAAACCTTTAGAAGAAAACATAAGGGAAATGCTTCATGACATTGAACTTTACAGTG
GGATTTGGACTCTCGATTTTGAGATTTTGGAAATCTTCTTTTGTATTCCCTTTACGAAGTACTGTAACTTGAAATGTCAC

GTGTCTTGGATATGATACCAAAAGCATGGACAATGAAAGAAAAAAAAACACATAGATTTCATCGAAGTAAAAACTTCTGT
CACAGAACCTATACTATGGTTTTCGTACCTGTTACTTTCTTTTTTTTTGTGTATCTAAAGTAGCTTCATTTTTGAAGACA

GCATTAAGTAACATTCTCAACAGAGTGAAAAGTCACAGAGGAAATATTTGCAAATCATATATCTGATAAGGGATTAATA-3′
CGTAATTCATTGTAAGAGTTGTCTCACTTTTCAGTGTCTCCTTTATAAACGTTTAGTATATAGACTATTCCCTAATTAT-5′

5′-AATTCTTTGGAAAAAAGACAAAGTTGAAATAATGTCAGAGAGAATACATCAGGAAAAAGGTTGAAAATGTAAGTTCTA
3′-TTAAGAAACCTTTTTTCTGTTTCAACTTTATTACAGTCTCTCTTATGTAGTCCTTTTTCCAACTTTTACATTCAAGAT

↑

TTAAACACAACATACTTAGAGGGGGAGAAAATCACAGAAGAGCCAGTTATGGCAGCTGAAAGAGGGGCAATGCTAAAGAC
AATTTGTGTTGTATGAATCTCCCCCTCTTTTAGTGTCTTCTCGGTCAATACCGTCGACTTTCTCCCCGTTACGATTTCTG

TCACATTTTTAATTTAAAAATCAGTGTTCCCTGGGCAGCAAGAAATTTTTGCCTATACTATATACAAAAAATTCATTCAA
AGTGTAAAAATTAAATTTTTAGTCACAAGGGACCCGTCGTTCTTTAAAAACGGATATGATATATGTTTTTTAAGTAAGTT

AGAGAATAAAAGACCTAAACCTGAGAGCTAAAACTCTAAAACCTTTAGAAGAAAACATAAGGGAAATGCTTCATGACATT
TCTCTTATTTTCTGGATTTGGACTCTCGATTTTGAGATTTTGGAAATCTTCTTTTGTATTCCCTTTACGAAGTACTGTAA

GAACTTTACAGTGGTGTCTTGGATATGATACCAAAAGCATGGACAATGAAAGAAAAAAAAACACATAGATTTCATCGAAG
CTTGAAATGTCACCACAGAACCTATACTATGGTTTTCGTACCTGTTACTTTCTTTTTTTTTGTGTATCTAAAGTAGCTTC

↓

TAAAAACTTCTGTGCATTAAGTAACATTCTCAACAGAGTGAAAAGTCACAGAGGAAATATTTGCAAATCATATATCTG-3′
ATTTTTGAAGACACGTAATTCATTGTAAGAGTTGTCTCACTTTTCAGTGTCTCCTTTATAAACGTTTAGTATATAGAC-5′

TSD: Chr12:77126920-77127284

EN cleavage: 5′-TTTTC/AA-3′

Supplemental Figure 5D

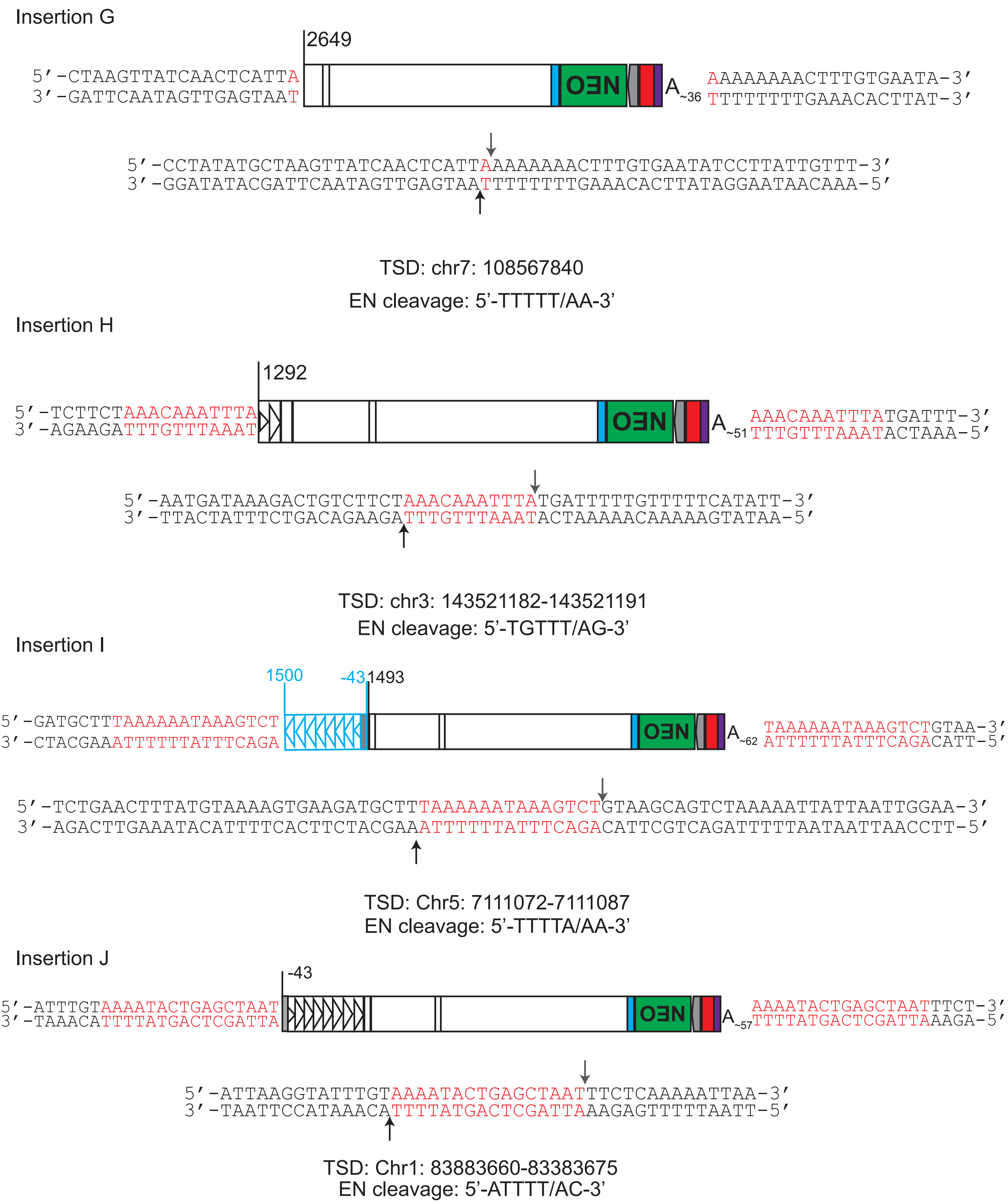

Supplement: Supplementary file 5 [file pgen.1012340.s005.pdf]
