# Supplementary material for: Revisiting the impact of synthetic ORF sequences on engineered LINE-1 retrotransposition
Source: PLoS Genet. 2026 Oct 16;22(10):e1012340. doi: 10.1371/journal.pgen.1012340 (PMC13705589; doi:10.1371/journal.pgen.1012340)

Biological Replicate 1

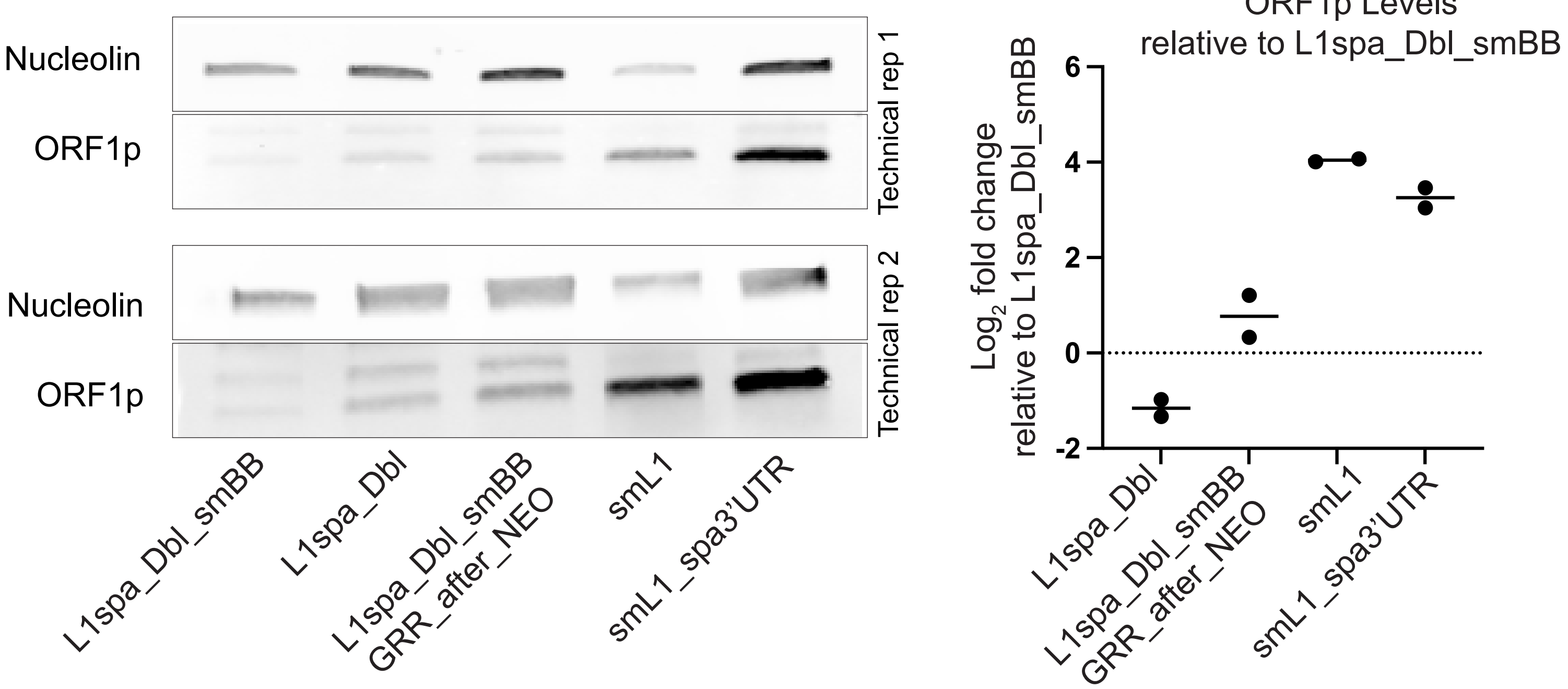

Biological Replicate 2

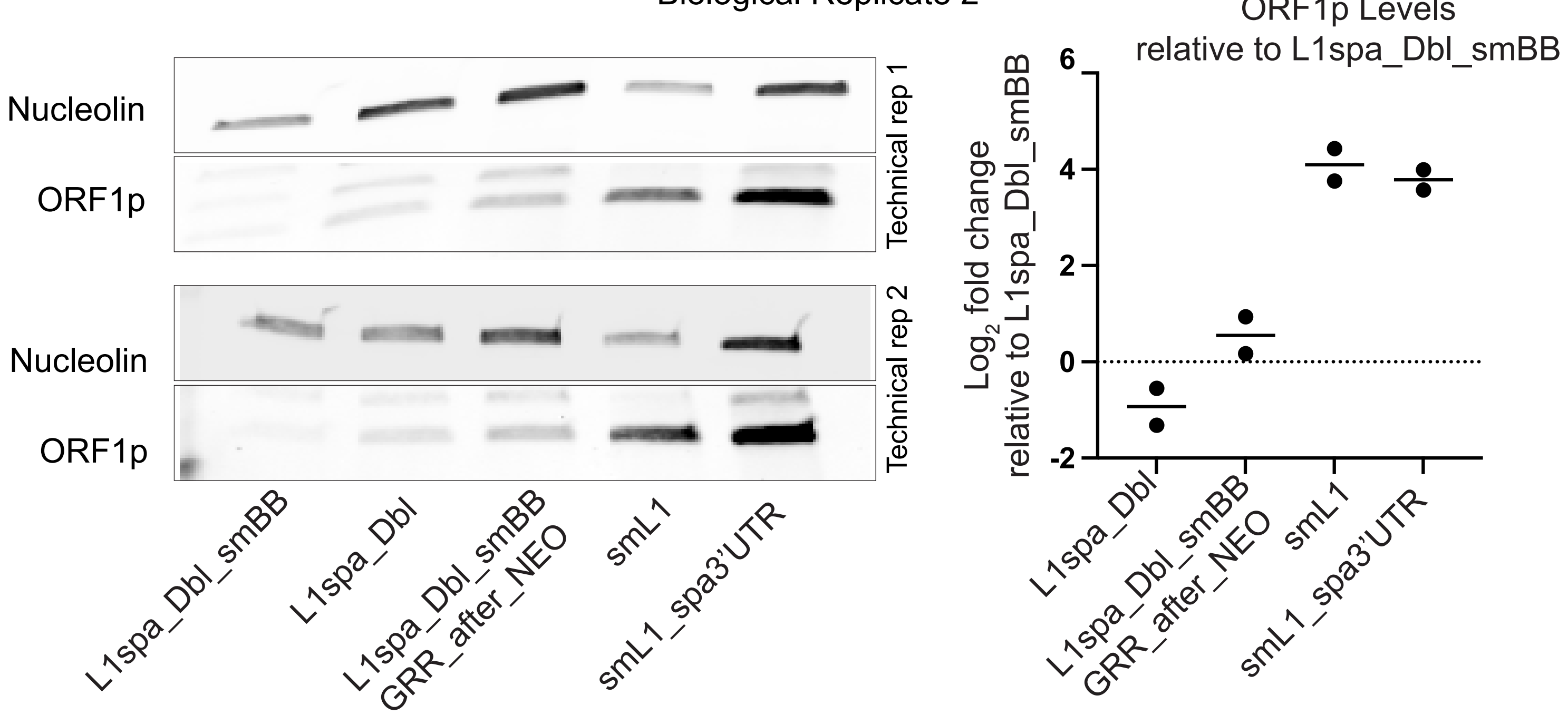

Biological Replicate 3

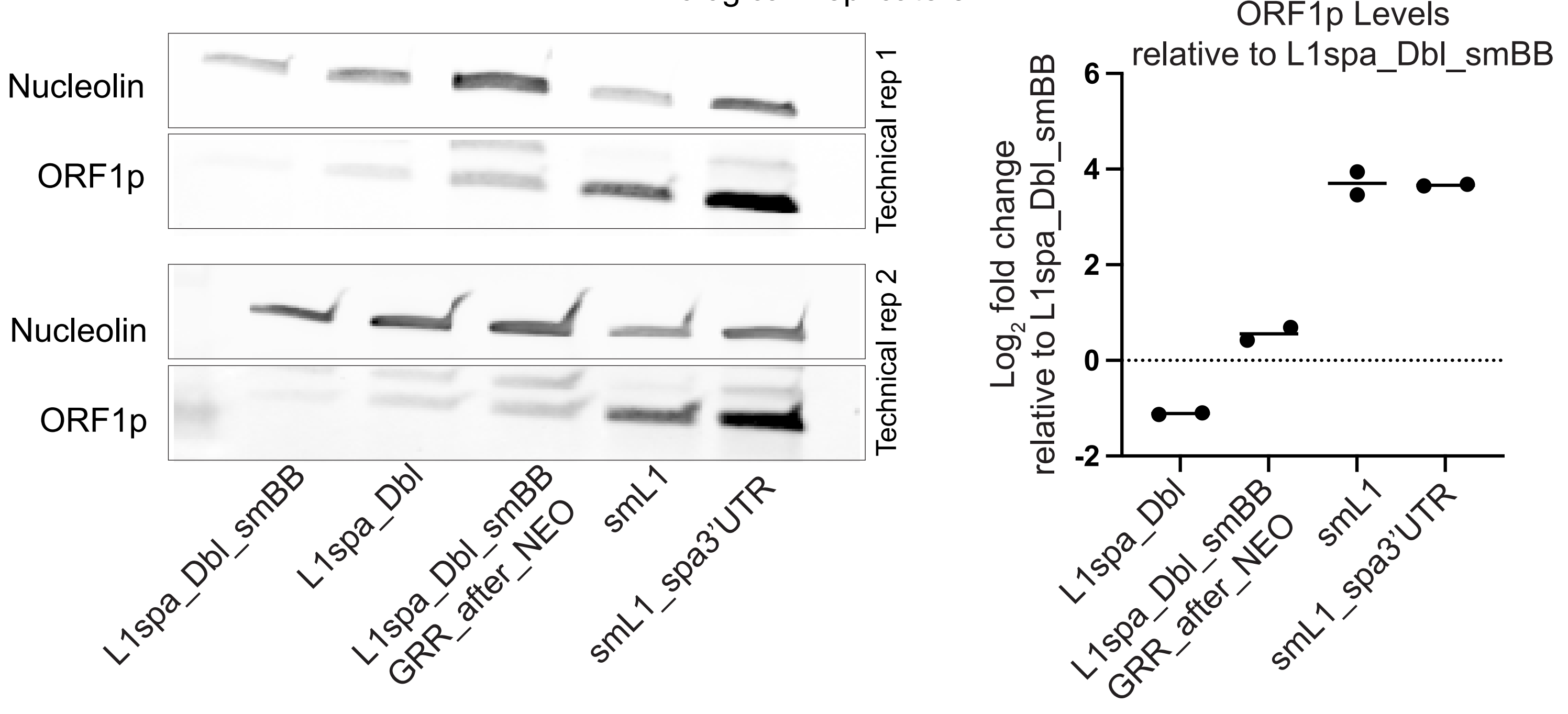

Supplement: Supplementary file 6 [file pgen.1012340.s006.pdf]
