# Supplementary material for: Revisiting the impact of synthetic ORF sequences on engineered LINE-1 retrotransposition
Source: PLoS Genet. 2026 Oct 16;22(10):e1012340. doi: 10.1371/journal.pgen.1012340 (PMC13705589; doi:10.1371/journal.pgen.1012340)
Supplement: Supplementary file 8 [file pgen.1012340.s009.pdf]

Figure 3A

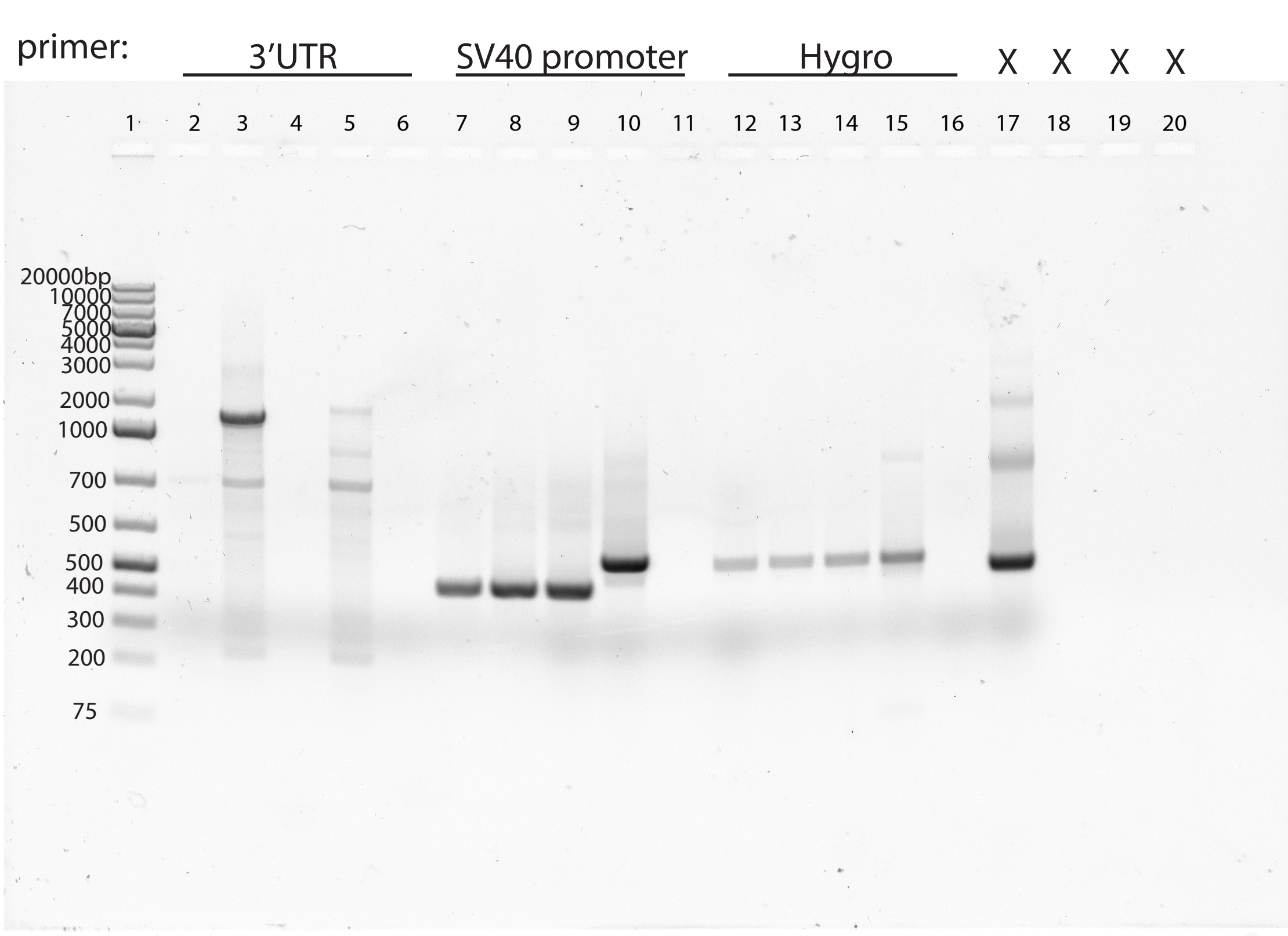

1. mw
2. L1spa_Dbl
3. L1spa_Dbl_smBB
4. Restore_3'UTR
5. GRR_after_NEO
6. no template
7. L1spa_Dbl
8. L1spa_Dbl_smBB
9. Restore_3'UTR
10. GRR_after_NEO
11. no template
12. L1spa_Dbl
13. L1spa_Dbl_smBB
14. Restore_3'UTR
15. GRR_after_NEO
16. no template
17. positive control (mouse thymus RNA, β-actin primer)
18. empty
19. empty
20. empty

Agarose gel was imged on a Typhoon FLA 9000 biomolecular imager.

Figure 3C (Same Gel as Sup Fig 6, Bio rep1, tech rep 1)

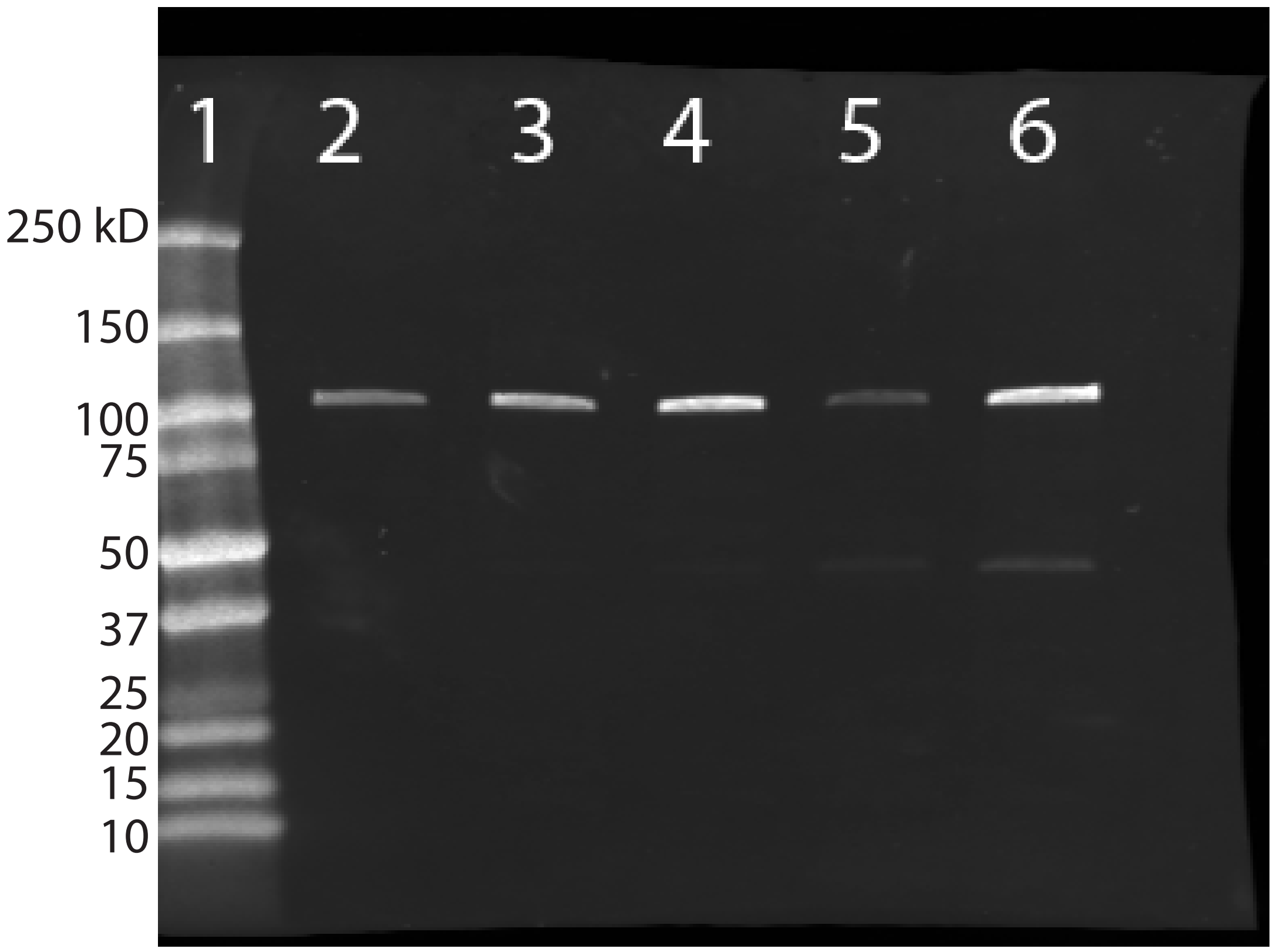

1. mw
2. L1spa_Dbl_smBB
3. L1spa_Dbl
4. L1spa_Dbl_smBB_GRRAN
5. smL1
6. smL1_spa3'UTR

Westerns blots were imaged on a LiCOR Odyssey CLx with the LiCOR Image Studio software.

Supplemental Figure 6

Bio rep 1, tech rep 1 (same gel as Fig 3C)

1 2 3 4 5 6

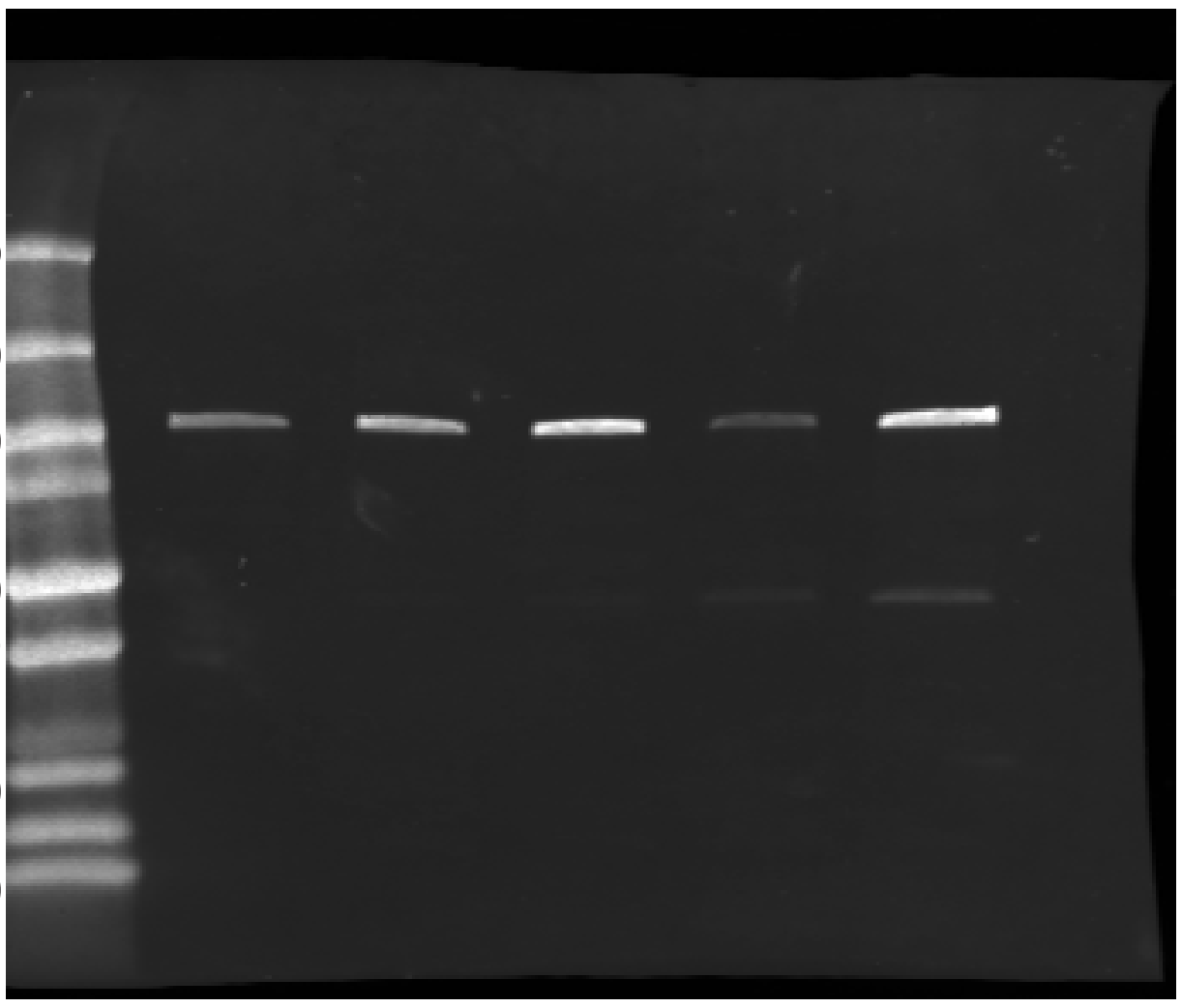

250 kD
150
100
75
50
37
25
20
15
10

Bio rep 1, tech rep 2

1 2 3 4 5 6

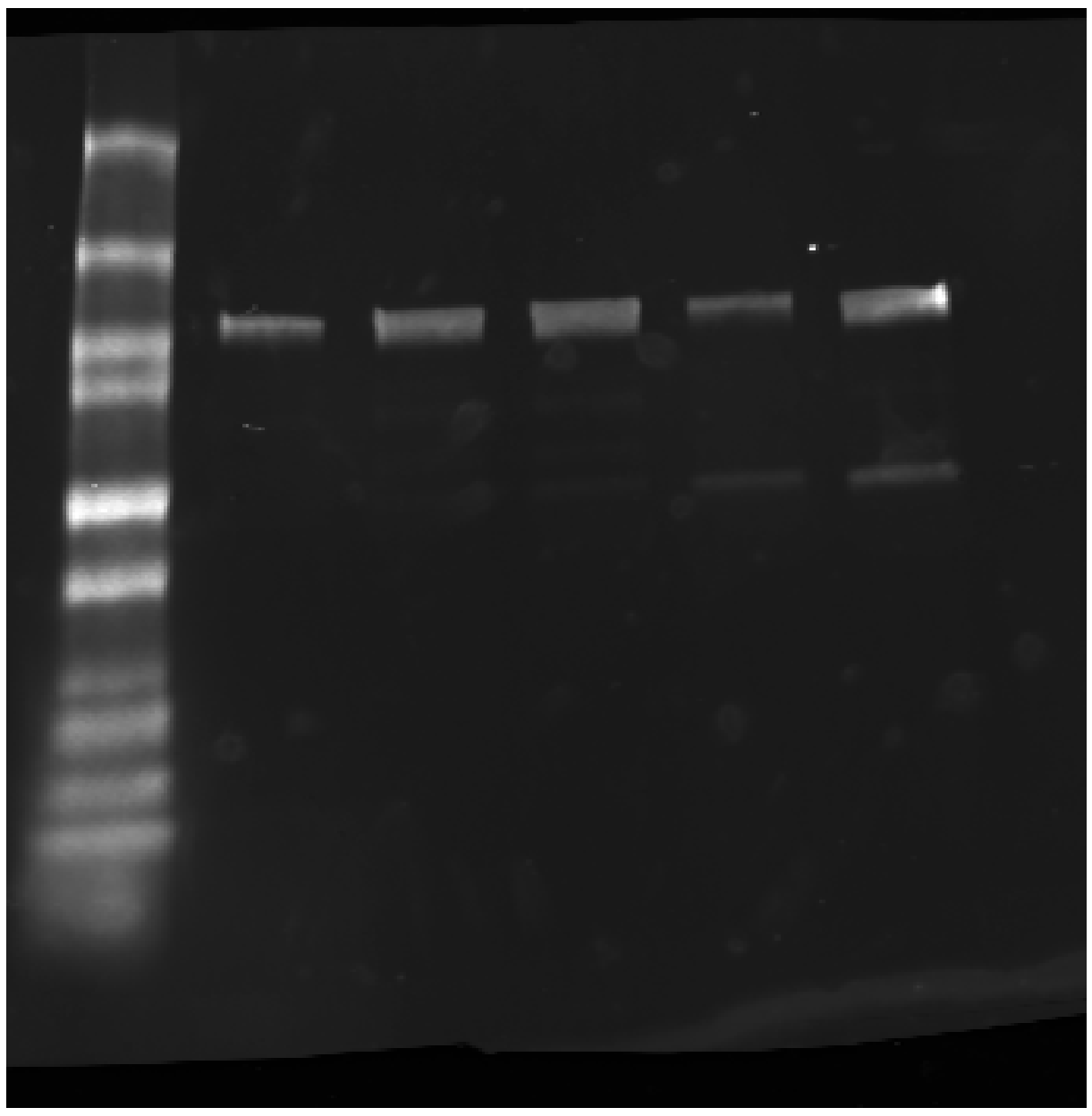

Bio rep 2, tech rep 1

1 2 3 4 5 6

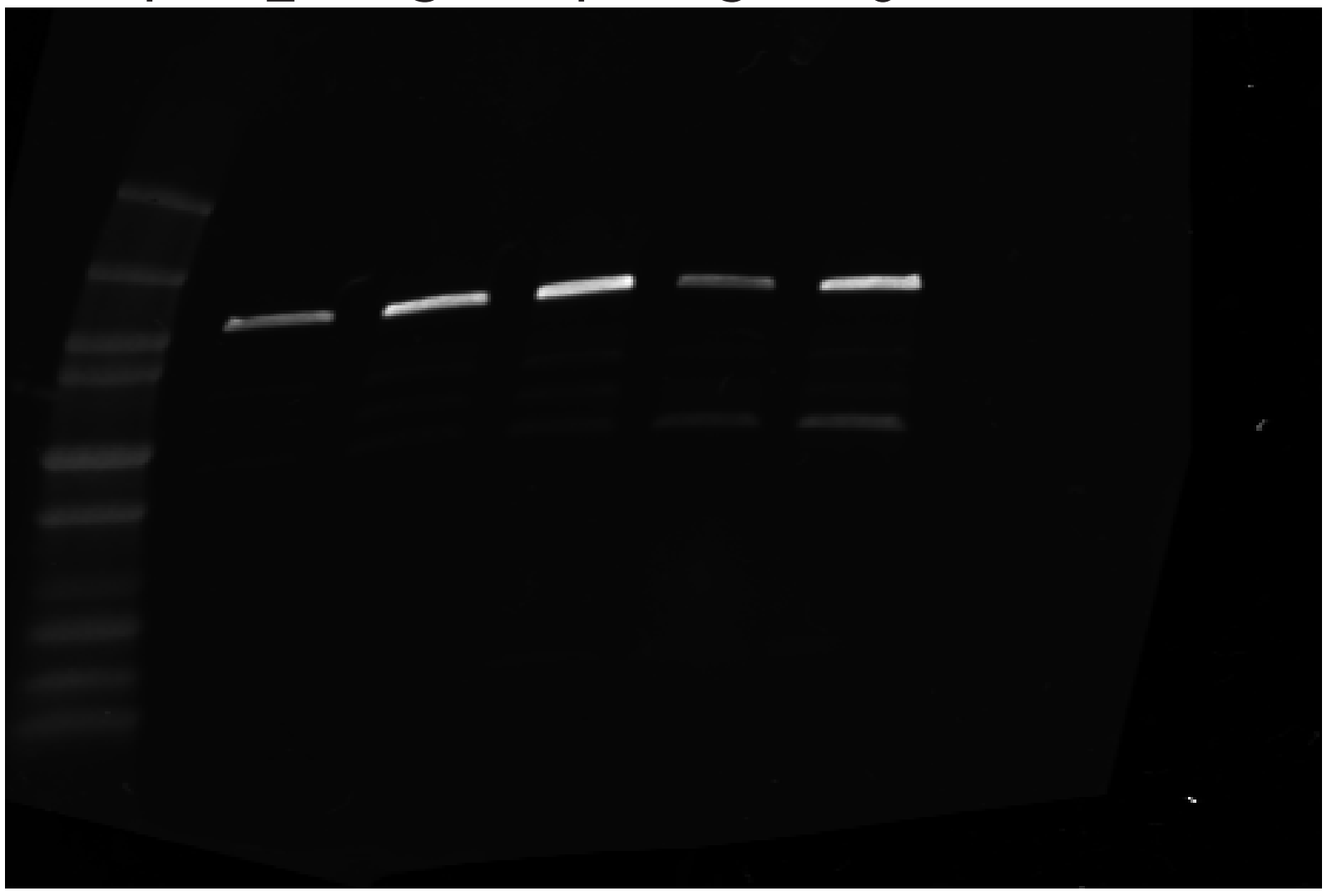

Bio rep 2, tech rep 2

1 2 3 4 5 6

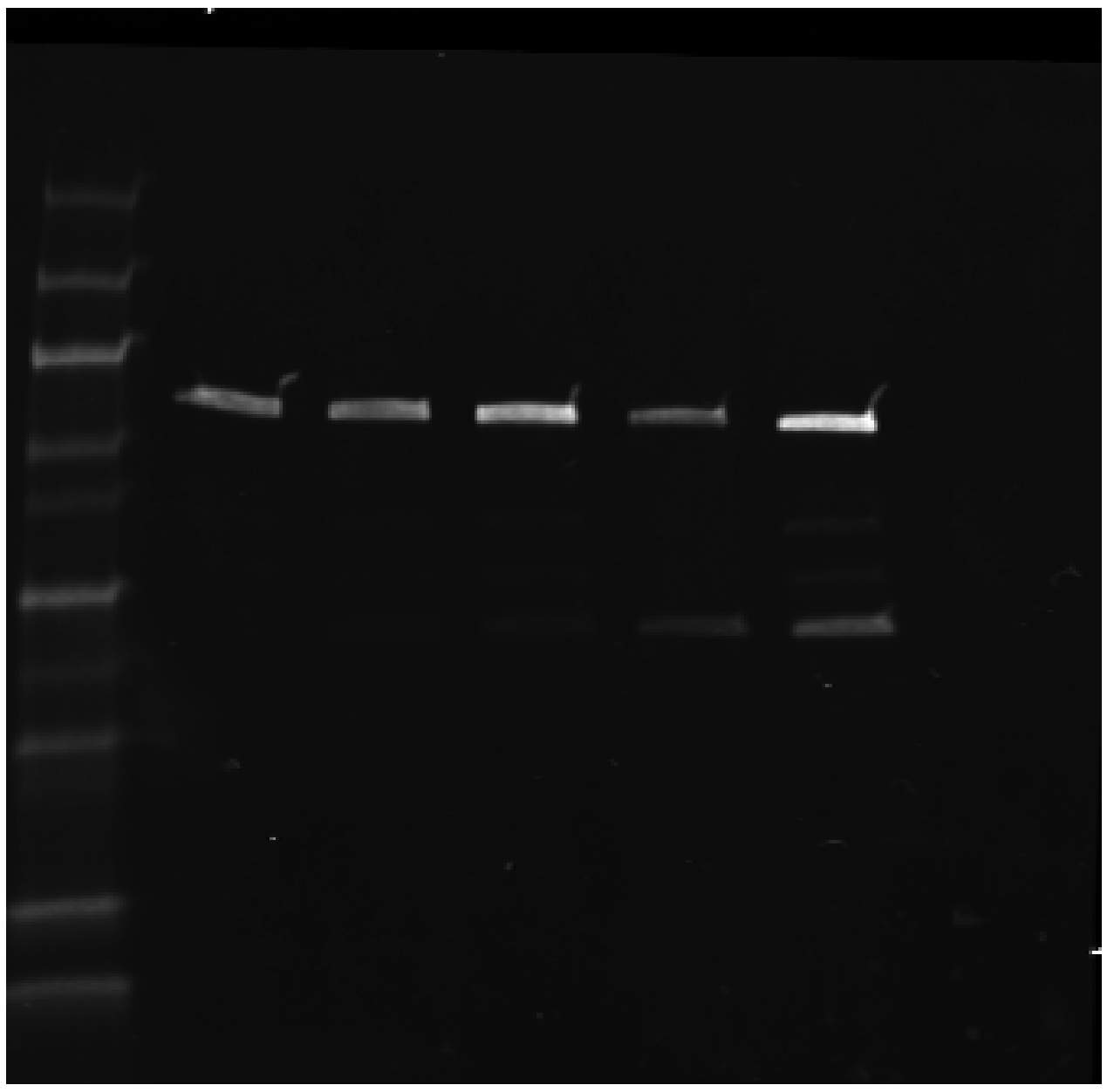

Bio rep 3, tech rep 1

1 2 3 4 5 6

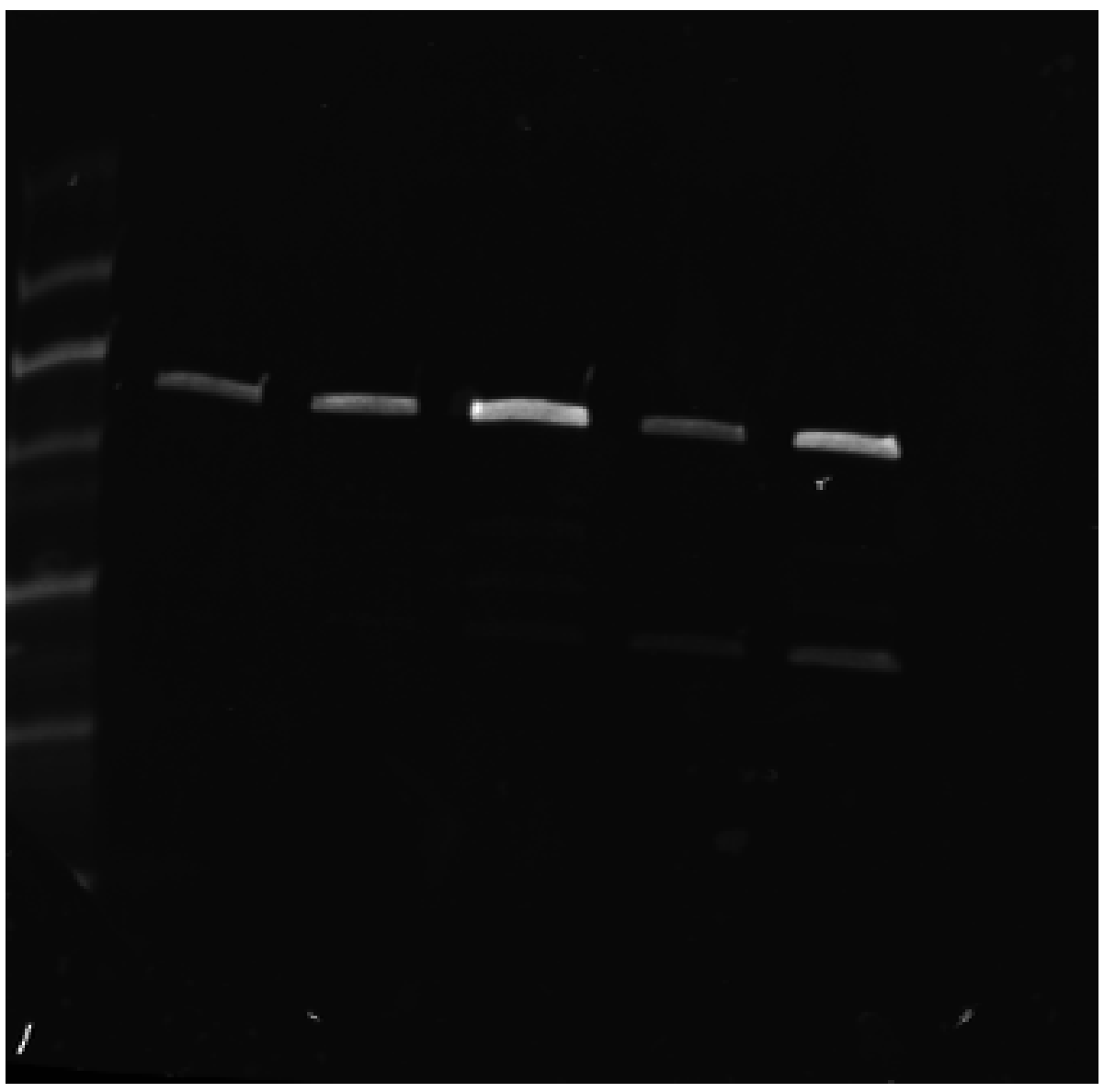

Bio rep 3, tech rep 2

1 2 3 4 5 6

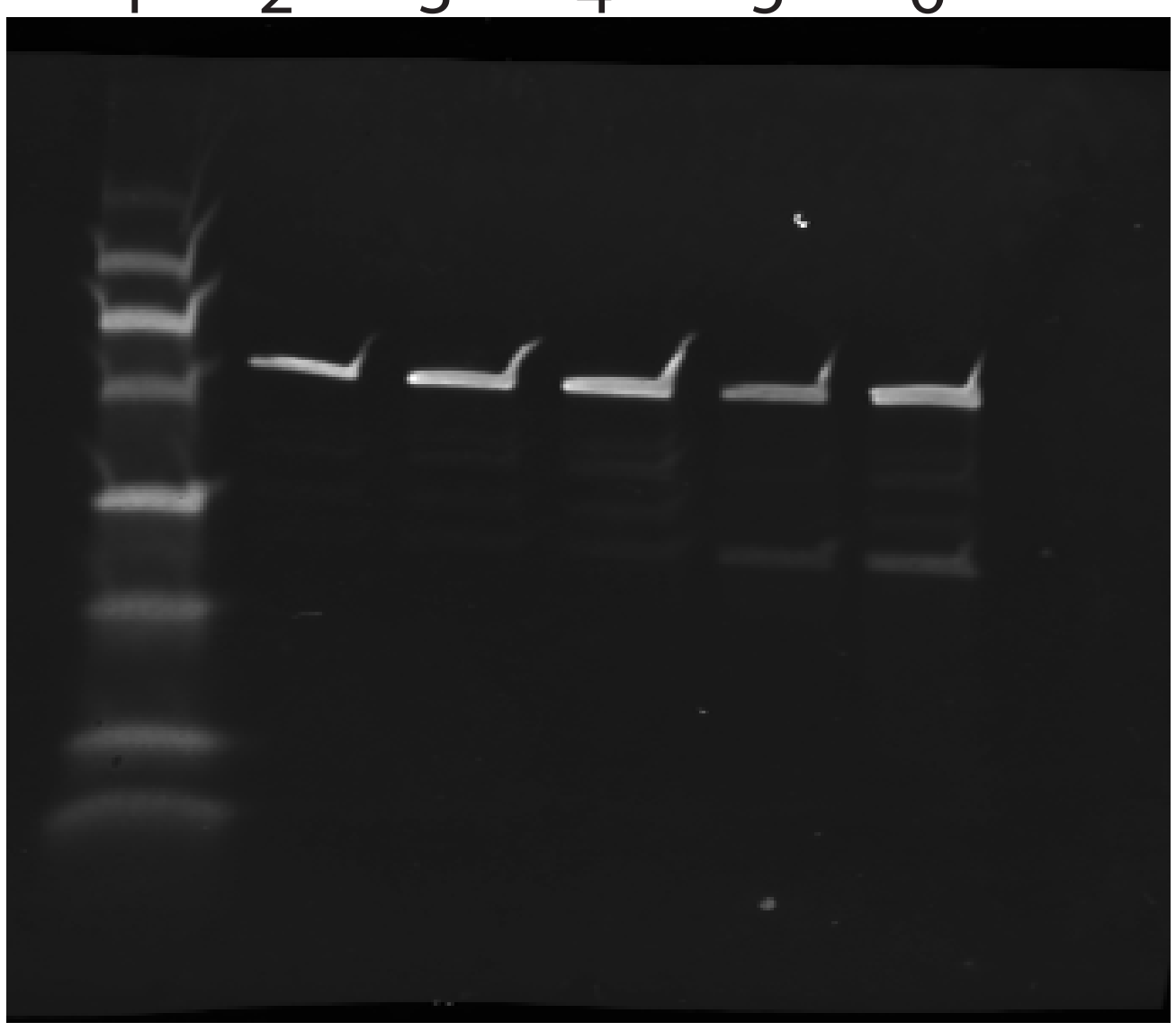

All gels:

1. mw
2. L1spa_Dbl_smBB
3. L1spa_Dbl
4. L1spa_Dbl_smBB_GRRAN
5. smL1
6. smL1_spa3'UTR

Westerns blots were imaged on a LiCOR Odyssey CLx with the LiCOR Image Studio software.

Supplemental Figure 7

Westerns blots were imaged on a LiCOR Odyssey CLx with the LiCOR Image Studio software.

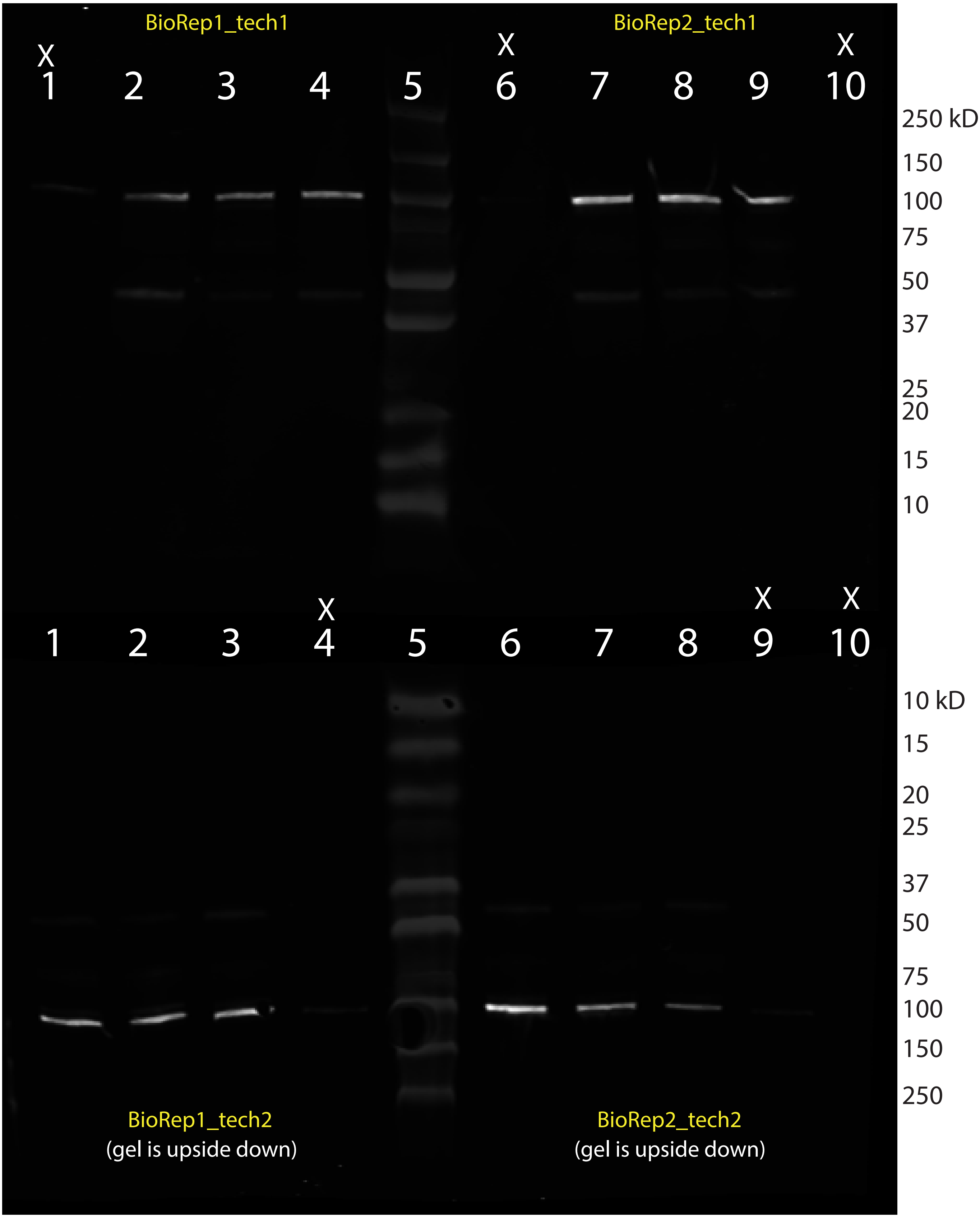

Upper gel
1. Untransfected
2. L1spa_Dbl_smBB_GRRAN
3. L1spa_Dbl_smBB_restore_3'UTR
4. L1spa_Dbl_smBB
5. mw
6. Untransfected
7. L1spa_Dbl_smBB_GRRAN
8. L1spa_Dbl_smBB_restore_3'UTR
9. L1spa_Dbl_smBB
10. empty well

Lower gel
1. L1spa_Dbl_smBB
2. L1spa_Dbl_smBB_restore_3'UTR
3. L1spa_Dbl_smBB_GRRAN
4. Untransfected
5. mw
6. L1spa_Dbl_smBB
7. L1spa_Dbl_smBB_restore_3'UTR
8. L1spa_Dbl_smBB_GRRAN
9. Untransfected
10. empty well

Supplemental Figure 7

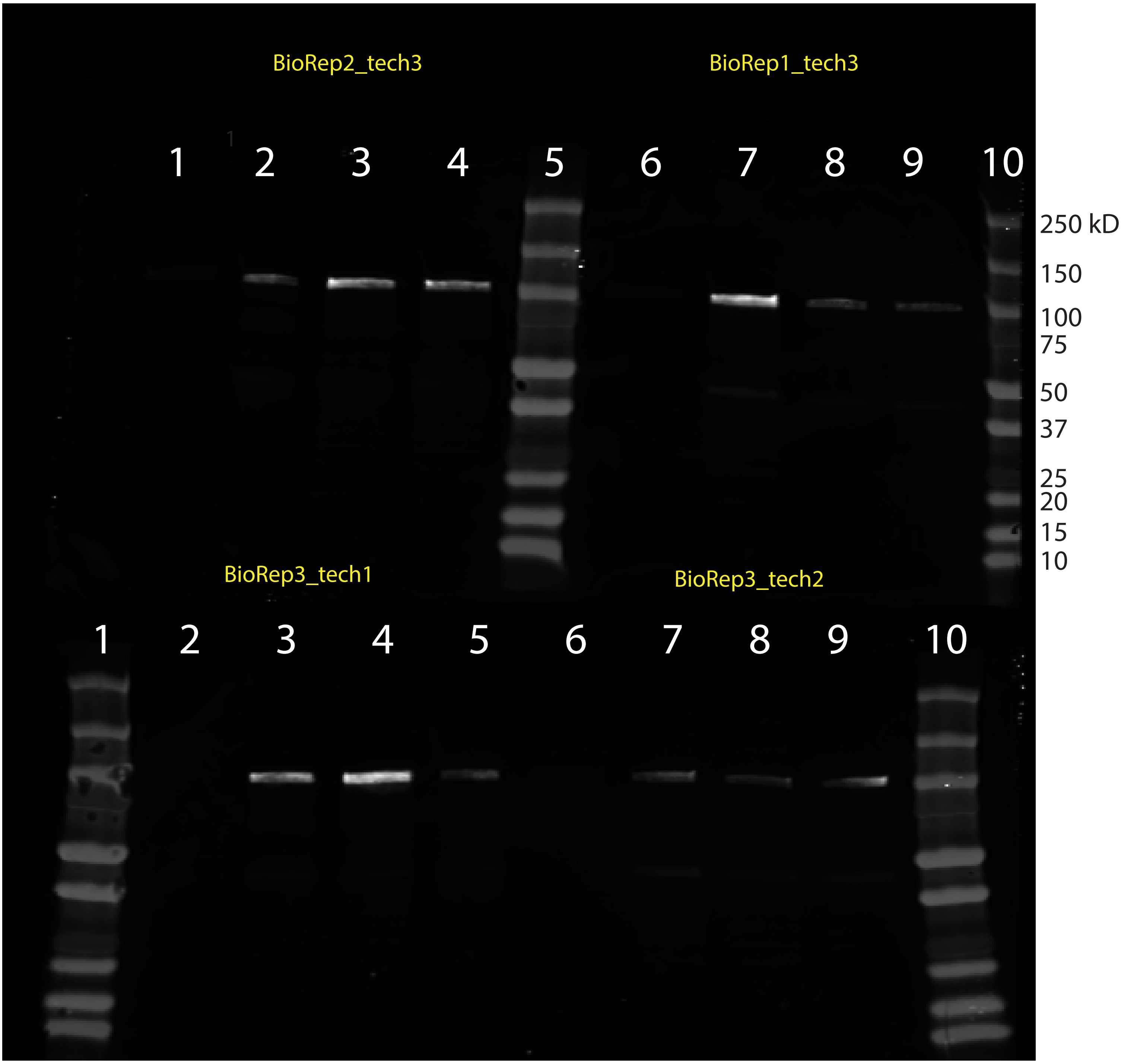

Upper gel
1. Untransfected
2. L1spa_Dbl_smBB_GRRAN
3. L1spa_Dbl_smBB_restore_3'UTR
4. L1spa_Dbl_smBB
5. mw
6. Untransfected
7. L1spa_Dbl_smBB_GRRAN
8. L1spa_Dbl_smBB_restore_3'UTR
9. L1spa_Dbl_smBB
10. mw

Lower gel
1. mw
2. Untransfected
3. L1spa_Dbl_smBB_GRRAN
4. L1spa_Dbl_smBB_restore_3'UTR
5. L1spa_Dbl_smBB
6. Untransfected
7. L1spa_Dbl_smBB_GRRAN
8. L1spa_Dbl_smBB_restore_3'UTR
9. L1spa_Dbl_smBB
10. mw

Westerns blots were imaged on a LiCOR Odyssey CLx with the LiCOR Image Studio software.
