# Supplementary material for: Revisiting the impact of synthetic ORF sequences on engineered LINE-1 retrotransposition
Source: PLoS Genet. 2026 Oct 16;22(10):e1012340. doi: 10.1371/journal.pgen.1012340 (PMC13705589; doi:10.1371/journal.pgen.1012340)
Supplement: Supplementary file 9 [file pgen.1012340.s010.pdf]

Overview: In response to the insightful reviewer comments on our manuscript “Revisiting the impact of synthetic ORF sequences on engineered LINE-1 retrotransposition”, we have generated several new retrotransposition indicator constructs and performed qRT-PCR and Western blot experiments to quantify engineered L1 RNA and ORF1 protein levels. The revised manuscript contains all of the data from the previous version, but has been substantially rearranged to allow a logical presentation of results. The previous Figure 4 now is Supplemental Figure 3, and the previous Supplemental Figure 3 is now Supplemental Figure 5. We have added new data to Figures 1 and 2, included two new panels in Figure 3, and have added three new Supplemental Figures, Supplemental Figure 4, Supplemental Figure 6, and Supplemental Figure 7.

We also performed long-read sequencing of all constructs used in this study; complete plasmid sequences are provided in Supplemental Table 1. Long-read sequencing revealed that smL1, smL1_mut2, and consequently all constructs containing the smL1 plasmid ‘backbone’ denoted as smBB (e.g., L1spa_Dbl_smBB) lack one complete $L1\_T_F$ monomer unit in the 5’UTR relative to pTN201/L1spa (Figure 1B). Across the comprehensive array of constructs generated for this study, this one-monomer deletion does not explain the observed differences in retrotransposition efficiency, L1 RNA levels, or L1 protein levels (Figure 1-3, Supplemental Figure 4). Moreover, we previously demonstrated that inclusion of a CMV promoter upstream of the mouse L1 5’UTR overrides differences in retrotransposition efficiency arising from varying numbers of 5’UTR monomer units (Gerdes et al. 2023).

We hope that these revisions will render our manuscript suitable for publication in PLOS Genetics. Our responses to individual reviewer comments are included below:

Reviewer #1: On this manuscript the authors revised data from a prominent study using a synthetic engineered mouse L1 retrotransposition (reduced ORF1 adenosine content) with increase levels of mRNA expression and retrotransposition when compared to the endogenous parental L1 element. The authors showed that reduction in ORF adenosine does not significantly change retrotransposition activity but the increased activity is due to a non synonymous ORF1 substitution (H159D), and deletino of a part of 3'UTR region. This observation explain why the same rationale was not mirrored when using a human synthetic L1 element, confirming that increase L1 mRNA levels are not rate-limiting determinant of retrotransposiion rates. I found the experiments very well-described and with all proper controls included. While the impact of the interpretation on the first publication is hard to measure, I applaud the authors to detect the initial artifact, their transparency, and for going deep to solve this issue. It is an example of how science should always be done.

We thank Reviewer 1 for their supportive remarks.

Reviewer #2: Review of Chan, Workman et al. entitled, “Revisiting the impact of synthetic ORF sequences on engineered LINE-1 retrotransposition” for PLoS Genetics.

Summary

The authors have conducted a nice study that clearly demonstrates that “apple to apple” comparisons of natural and synthetic mouse L1s that differ only in their respective adenosine contents retrotranspose a very similar levels in cultured cells. These data, in the author’s words, “…prompt a reevaluation of synthetic L1 activity and suggest native mouse L1 mobility has in some cases been underestimated in engineered retrotransposition assays.

Overall, this is a very well written paper and clearly demonstrates that two ORF1 amino acid differences and the presence/absence of a guanosine rich region (GRR) in the L1 3’ untranslated region

(UTR) are responsible for the previously reported retrotransposition efficiency differences between a natural L1 element (L1spa) and a synthetic L1 element (L1Sm).

In truth, previous publications (e.g., PMID: 21320307) alluded to the possibility that decreasing the adenosine content of L1 RNA does not result in increased retrotransposition of an engineered synthetic human L1 relative to its natural counterpart. However, this manuscript has done a very nice job at identifying sequence changes in the synthetic mouse L1 (L1Sm) relative to its supposed natural L1 (L1 spa) that account for the previously reported 200-fold difference in retrotransposition efficiency.

Major Comments

Ideally, it would be nice if the authors tested whether their true reassembled version(s) of L1spa and their L1Sm counterpart show differences in steady state RNA expression and ORF1p/ORF2p levels in HeLa-JVM cells, as it would convincingly test whether changing the adenosine content of L1 RNA results in L1 expression changes, but not a difference in retrotransposition. Also, it would be interesting to learn how the repositioning of the GRR relative to the retrotransposition cassette inhibits retrotransposition. The inclusion of the above (or similar) experiments would improve the "mechanistic" insight and biological novelty of the study.

We thank Reviewer 1 for their constructive comments. We have expanded our investigations into the mechanism by which the presence and position of the GRR impacts retrotransposition efficiency to include a qRT-PCR based quantitation of L1 RNA levels, and we have quantified L1 ORF1p expression via Western blot. Our qRT-PCR results are shown in Supplemental Figure 4, and described on pg. 9-10, lines 212-239 of the revised manuscript:

"To investigate the impact of the GRR presence and position upon engineered L1 RNA levels, we performed qRT-PCR on RNA isolated from HeLa cells transfected with the constructs L1spa_Dbl, L1spa_Dbl_SMBB, L1spa_Dbl_SMBB_GRR_after_NEO, smL1, or smL1_spa3'UTR. We employed primer pairs targeting the non-monomeric region of the 5'UTR, the L1spa GRR, and Orf1 and Orf2 sequences of L1spa and smL1. We also targeted a portion of the pCEP4 SV40 polyadenylation signal (SV40pA) that lies upstream of the AAUAAA motif and cleavage site and therefore is expected to be incorporated into the full-length L1 transcriptional unit (Supplemental Figure 4A). The RNA levels for each target were calculated relative to expression of the endogenous housekeeping gene ribosomal protein S18 (*Rps18*).

Across all target sequences, smL1 RNA levels were significantly higher than any of the natural mouse L1 constructs ($p<0.05$, one-way ANOVA with Tukey's multiple comparisons test; Supplemental Figure 4, B-E), consistent with previous reports of elevated RNA expression from synthetic L1 constructs (1, 43). Among the natural mouse L1 constructs, RNA levels for L1spa_Dbl_smBB were consistently elevated compared to L1spa_Dbl and L1spa_Dbl_smBB_GRRAN. Although these differences did not reach statistical significance, this result suggests that inclusion of the GRR within the L1 transcriptional unit may reduce L1 RNA levels. Indeed, RNA levels for smL1_spa3'UTR were also diminished relative to smL1 for all target sequences, reaching statistical significance for the Orf2 ($p<0.001$) and 5'UTR ($p<0.05$) primer pairs. Primers targeting the GRR sequence itself gave no signal for the GRR-lacking L1spa_Dbl_smBB and smL1 constructs, as expected (Supplemental Figure 4F). GRR RNA levels were lower for L1spa_Dbl compared to L1spa_Dbl_smBB_GRRAN and smL1_spa3'UTR; though not statistically significant, this result suggests that L1spa GRR transcription may be influenced by position within the expression construct or the presence of upstream synthetic ORFs. The above results must be interpreted with the caveat that qRT-PCR signal for short stretches of L1 RNA sequence does not

necessarily reflect the abundance of full-length L1 mRNA molecules capable of undergoing retrotransposition. Nevertheless, we conclude that synthetic ORFs and the presence and position of the GRR sequence within the L1 transcriptional unit can modulate engineered L1 RNA levels, but this modulation does not correlate with or explain the observed differences in retrotransposition efficiency (compare Figure 2 and Supplemental Figure 4B-F)."

Our Western blot results are shown in Figure 3C-D and Supplemental Figures 6 and 7, and are described on pg. 10, lines 240-257:

" Finally, we investigated the impact of GRR presence and position upon L1 ORF1 protein expression levels from natural and synthetic mouse L1 constructs. We transfected HeLa cells with L1spa_Dbl, L1spa_Dbl_SMBB, L1spa_Dbl_SMBB_GRRAN, smL1, and smL1_spa3'UTR and performed Western blots on HeLa cell extracts using an antibody specific to mouse L1 ORF1p (54) and an antibody to nucleolin as a loading control. Among three biological replicate experiments, mean nucleolin-normalized L1spa_Dbl ORF1p levels were ~2-fold lower compared to L1spa_Dbl_smBB, while L1spa_Dbl_smBB_GRRAN ORF1p levels were increased over L1spa_Dbl_smBB by ~1.5-fold (Figure 3C-D, Supplemental Figure 6). L1 ORF1p levels from smL1 and smL1_spa3'UTR were elevated ~15-fold and ~12-fold, respectively, relative to L1spa_Dbl_smBB, consistent with the previously reported increase in protein expression from synthetic L1 constructs (1, 43). To rule out the absence of a Kozak sequence on the L1spa_Dbl ORF1p initiation codon as the reason for diminished ORF1p levels compared to the other Kozak-containing constructs (Figure 2), we verified in a separate experiment that ORF1p levels from L1spa_Dbl_smBB_Restore_3'UTR, which is identical to L1spa_Dbl_smBB except for the inclusion of the L1spa GRR upstream of the NEO cassette (Figure 2A) also are ~2-fold lower than L1spa_Dbl_smBB (Supplemental Figure 7). Taken together, our results indicate that placement of the GRR upstream, but not downstream, of the NEO cassette diminishes ORF1p expression, implicating reduced ORF1p levels in the position-dependent effect of the GRR upon natural mouse L1 retrotransposition efficiency."

Based on these results, we speculate on the relationship between L1 RNA and protein levels and observed retrotransposition efficiency in the Discussion (pg. 11-12, Lines 268-307):

"Thus, while both human and mouse synthetic L1 ORFs yield increased L1 RNA levels relative to native ORF sequences, this elevated expression alone does not effect a proportionate increase in retrotransposition (Figure 1, Figure 2, Supplemental Figure 4)(1, 43). This result is consistent with the modest increase in retrotransposition achieved by augmenting L1 expression with a strong heterologous promoter (19). While regulation of L1 expression is a critical mechanism for limiting endogenous retrotransposition (67), our results suggest that in contexts where L1 expression surpasses a certain threshold, L1 mRNA levels are not the limiting determinant of retrotransposition efficiency. Moreover, our findings emphasize that deleting or reconfiguring portions of the L1 sequence when generating engineered reporter constructs can dramatically impact detected retrotransposition efficiency and complicate interpretation of experimental results.

Our results suggest that the molecular mechanism by which deleting or repositioning the L1spa GRR impacts natural mouse L1 retrotransposition efficiency involves modulation of ORF1 protein levels. Relative to a natural mouse L1 construct lacking the GRR (L1spa_Dbl_smBB), placement of the GRR upstream of the NEO cassette (L1spa_Dbl or L1spa_Dbl_smBB_restore_3'UTR) diminishes ORF1p levels 2-fold, and its placement downstream of the NEO cassette, proximal to the 3' end of the L1

transcriptional unit (L1spa_Dbl_smBB_GRRAN), increases ORF1p levels ~1.6-fold (Figure 3C-D, Supplemental Figure 6, Supplemental Figure 7). The position-dependent impact of the GRR on natural mouse L1 ORF1p levels contrasts with our results for L1 RNA levels, which appear to be diminished by the inclusion of the GRR regardless of its placement within the construct (Supplemental Figure 4). ORF1p levels from a synthetic mouse L1 construct lacking the GRR (smL1) are elevated ~15-fold over the congruent natural L1 construct (L1spa_Dbl_smBB), consistent with enhanced protein expression from codon-optimized ORFs, and are modestly reduced by inclusion of the GRR upstream of the NEO cassette (smL1_spa3'UTR)(Figure 3C-D, Supplemental Figure 6). Critically, all the above-described constructs retrotranspose with similar efficiency except for L1spa_Dbl and L1spa_Dbl_smBB_Restore_3'UTR, which retain only ~20% relative retrotransposition efficiency (Figure 1-2, Supplemental Figure 3).

We propose that increased ORF1p levels effect a proportionate increase in retrotransposition only up to a certain threshold, which for a natural mouse L1 is exceeded when the GRR is deleted or placed downstream of the NEO cassette. Consistently, due to the dramatically elevated levels of smL1 ORF1p expression achieved via codon optimization, the diminution of ORF1p levels when the GRR is placed upstream of the NEO cassette (smL1_spa3'UTR) has no impact on retrotransposition efficiency (Figure 2A, Figure 3C-D, Supplemental Figure 6). This model also is consistent with the inability of GRR deletion or repositioning to fully overcome the negative effect of the H159 variant in L1spa ORF1p (Figure 2B). While L1spa_smBB and L1spa_smBB_GRRAN likely produce more ORF1p than L1spa_Dbl, the ORF1p produced is impaired for nucleic acid chaperone activity and therefore efficient retrotransposition, rendering the H159 variant largely epistatic to the deleted or repositioned GRR. The mechanism by which the presence and position of the GRR sequence influence L1 ORF1p translation or steady-state levels is a topic for future investigation. In addition, the impact of the GRR on L1 ORF2p protein levels remains to be elucidated."

Minor Comments

Abstract: Long Interspersed Element-1 (I think including the hyphen is standard in the field).

We have amended this oversight.

Page 2, line 49: Please include PMID: 8945518, it is the first reference to show that mutations in ORF1 and ORF2 strongly inhibit L1 retrotransposition.

This reference has been included.

Page 2, line 52: The LINE-1 EN consensus cleave sequence is: 5'-TTTTT/AA as reported in PMID: 30955886; that reference should be cited in addition to ref. 28.

This reference and the updated consensus cleavage sequence have been included. We also have updated the L1spa_Dbl_smBB_GRRAN insertion structures (Figure 3B and Supplemental Figure 5A-D) to display this extended EN consensus cleavage sequence.

Page 3, line 58 (and following text): Please consider citing PMID: 26895052 in addition to ref. 37; it is more current.

This reference has been added to the text as appropriate.

Page 7, line 120: please make the text and figure congruent with respect to the construct name

L1spa_Dbl_smBB_GRRAN; it took me a while to reconcile the name difference of the text construct and the one shown in Figure 2.

The text has been amended as follows, and the construct is consistently referred to as L1sp_Dbl_smBB_GRRAN in the manuscript and figures (Lines 163-168):

"To evaluate directly how placement of the GRR relative to the NEO cassette impacts retrotransposition efficiency of a natural mouse L1, we placed the L1spa GRR into L1spa_Dbl_SMBB downstream of NEO but upstream of the pCEP4 vector SV40 polyadenylation signal, to generate L1spa_Dbl_smBB_GRR_after_NEO, hereafter abbreviated as L1spa_Dbl_SMBB_GRRAN. "

Page 7, end of page: can the authors consider listing the putative L1 EN insertion sites in the main text (or Figure 3); I know it is mentioned explicitly in the supplement, but including these data in the main text would be a welcome addition to the paper.

The putative EN cleavage site for each insertion has been added to the right of the insertion structure in Figure 3B, and the text has been updated to include insertion at the EN motif as a hallmark of retrotransposition (Lines 206-211):

"Like smL1 insertions previously characterized in HeLa cells (1), all 10 L1spa_Dbl_smBB_GRRAN insertions bore the hallmarks of *bona fide* retrotransposition events including variable-length TSDs and insertion at sites resembling the L1 EN cleavage consensus motif 5'-TTTTT/AA-3' (Supplemental Figure 5, Supplemental Table 1)(30)"

Page 16, line 204: the authors may consider citing PMID: 20602998, as it alluded to the idea that L1s are jumping as fast as they can to outpace host restriction.

This reference has been included, we appreciate the excellent suggestion.

Reviewer #3: L1 retrotransposons are A/T rich sequences, and this composition has been presumed to limit the transcription of L1s. Indeed, previous work from one of the authors made many synonymous A/T to G/C changes in natural L1 sequences from mouse and found highly increased steady state transcript levels and highly increased rates of in vitro retrotransposition. In contrast, similar A/T decreasing synonymous changes in a human L1 increased transcript levels but had only a moderate effect on in vitro retrotransposition. This manuscript by Chan, Workman, Faulkner, Richardson and colleagues investigates this discrepancy between the human L1 and mouse L1 phenotypes associated with decreasing A/T content by deconvoluting the effects of the synonymous A/T mutations from nonsynonymous mutations and changes in construct architecture that were included but not controlled for in the creation of the hyperactive synthetic mouse L1, smL1, aka ORFeus-mm.

The authors find that one (and maybe two) nonsynonymous mutations in ORF1 relative to the non-A/T-optimized/natural L1 (L1spa, the causative L1 mutation in the spastic mouse phenotype) have some effect on retrotransposition, increasing retrotransposition of L1spa about 3-fold; reversion mutations in the synthetic smL1 decrease retrotransposition by approximately 40%. Next, the authors test the effect of a G-rich region (GRR, which is found naturally in L1s, but was removed in the cloning of the A/T-optimized smL1 construct) in combination with the adjacent 3'UTR sequence which contains 19 SNPs and 2 single-nucleotide deletions in smL1 relative to the natural L1. By swapping the 3'UTR from the synthetic smL1 (19 SNPs + 2 deletions + deleted GRR) into the natural L1spa, the natural L1 surprisingly retrotransposes at a very high rate, comparable to the A/T-optimized synthetic

smL1. The authors conclude that the effect of A/T optimization is minimal and the activity gains of the synthetic mouse L1 are instead due mostly to the GRR deletion in the 3'UTR. Further, the data show that the inhibitory effect of the GRR is likely an 'artifactual' interaction with the reporter gene sequence inserted 3' of the L1 3'UTR but before the transcription terminator/polyA signal. It seems the GRR has a negligible or slightly enhancing effect when placed near the end of the transcript, but inhibits when there is a stretch of DNA between the GRR and the end of the transcript. This finding has important implications for a large number of papers and groups who have used these constructs to test the activity of mouse L1s. In this way, the location of the GRR before the reporter gene has likely resulted in false negative measurements -- removal of the GRR or placement of the GRR after the reporter gene sequence may result in measurable or high retrotransposition activity of the tested L1.

The authors make several other conclusions about the necessity or sufficiency of various other changes to the L1 construct to modify retrotransposition activity that are not completely supported by the data, but these can likely be addressed with text changes or inclusion of some controls that the authors may even have already tested. In addition, this manuscript describes a lot of data and some modification of the graphical presentation of the data would greatly accommodate the reader's ability to understand this highly iterative data. Overall, this is an impactful finding, as many groups have used these constructs for many different purposes, and the core conclusion about the effect of GRR placement is well supported.

Major concerns:

1) The conclusions about the effects of each variant/mutation should be softened or supported with more variants and controls in several places:

a) (line 22) "We find that the combined effect of a single amino acid change and the GRR 3’UTR deletion, rather than synthetic ORF sequences, accounts for the increase in smL1 retrotransposition efficiency over L1spa." and (line 36) "two coding changes and partial deletion of the mouse L1 3’UTR sequence which occurred during construction of the synthetic mouse L1 reporter actually are responsible for the increased jumping of this construct.

The effect of the mutations at positions 53 and 159 are clear in the L1spa background, but are not tested for sufficiency in the other backgrounds. For example, does smL1_H159D behave the same as smL1_Dbl?

We had already created and tested the two single amino acid changes in smL1 (smL1_D159H and smL1_D53G), but we left them out of the original submission for the sake of simplicity. We now have included both constructs in Figure 1C and amended the text as follows (pg. 5, Lines 111-114):

“We likewise generated smL1_D53G, smL1_D159H, and smL1_Dbl (D53G and D159H), and found that while smL1_D53G retrotransposed with similar efficiency to smL1, smL1_D159H and smL1_Dbl exhibited a ~40% reduction in retrotransposition efficiency relative to smL1 (Figure 1C).”

Formally, there could be interactions between the amino acids at positions 53/159, the GRR presence, the GRR placement, and the sequence of the non-GRR-3'UTR. These interactions could be tested with many permutations, but are not necessary for the core finding of the paper. Instead, the authors should appropriately qualify each statement about causation of a change.

We thank Reviewer 3 for their insightful comments. Throughout the revised manuscript we have softened our wording to avoid over interpretation of results.

-line 22 should read "effect of two amino acid changes", as only the double mutation is tested in figures 2-4; the authors don't test the single amino acid mutation H159D plus GRR changes, only DD plus GRR changes.

We have changed the wording to "We find that the combined effect of the altered ORF1p amino acid sequence and the GRR 3'UTR deletion, rather than synthetic ORF sequences, accounts for the increase in smL1 retrotransposition efficiency over L1spa." (pg. 1, Lines 22-24).

It could be useful to mention why the authors proceed with the DD variant instead of GD.

We consider L1spa_Dbl as the "wild-type" L1_T_F sequence and used it as the basis for comparison in subsequent experiments because the L1_TF consensus sequence and all active L1_TF elements evaluated to date (apart from L1spa) contain aspartic acid at ORF1p positions 53 and 159. L1spa_Dbl and smL1 also have identical ORF1p amino acid sequences, making L1spa_Dbl rather than L1spa_H159D the appropriate control for subsequent experiments. We now have stated this explicitly in the text (pg. 5, Lines 117-121):

"The G53 and H159 variants apparently are restricted to L1spa and closely related elements, as the L1_TF consensus contains both D53 and D159 (6-8, 46-48). As L1spa_Dbl and smL1 have identical ORF1p amino acid sequences, we used L1spa_Dbl rather than L1spa_H159D as the basis for comparison to smL1-derived constructs in subsequent experiments."

-the necessity of the 53/159 mutations to the full activity of L1spa_Dbl_smBB is not tested. The activity change could happen with just GRR after Neo, keeping G53 and H159.

We thank Reviewer 3 for the interesting suggestion and we have tested this possibility by generating the constructs L1spa_smBB and L1spa_smBB_GRRAN, which contain the original amino acid sequence of L1spa (G53 and H159), but have the GRR sequence deleted entirely or located downstream of the NEO cassette, respectively. These constructs retrotransposed very slightly more efficiently than L1spa, but did not approach the activity of L1spa_Dbl_smBB or L1spa_Dbl_smBB_GRRAN. This data now is included in Figure 2B. As the H159 residue in L1spa previously has been demonstrated to diminish ORF1p chaperone activity (Martin et al. 2005, Martin et al. 2008), we reason that this mutation is epistatic to the effects of the placement of the GRR sequence, and that moving the GRR cannot compensate for a sub-functional ORF1p protein. We now have included these data in Figure 2B and address them with the following text (pg. 7, Lines 171-178):

"We also asked whether removing or relocating the GRR can enable efficient retrotransposition of the original L1spa element. We generated a construct containing the original L1spa ORF1p sequence (G53 and H159) but with the GRR deleted (L1spa_SMBB) or placed downstream of the NEO cassette (L1spa_SMBB_GRR_after_NEO). Both constructs retrotransposed at only ~20% the efficiency of L1spa_Dbl_smBB or L1spa_Dbl_smBB_GRRAN, constituting a slight improvement over L1spa (Figure 2B). We conclude that deletion of the GRR cannot fully compensate for the previously reported (46, 47) effects of H159 upon L1spa ORF1p nucleic acid chaperone activity and retrotransposition efficiency."

b) (line 175) "Here we demonstrate that synthetic reduction in mouse L1 ORF adenosine content has little impact on retrotransposition efficiency, at least in HeLa cells"

The data don't explicitly test the effect of A/T optimization alone, only in combination with the smL1 3'UTR which has the GRR deletion. For example, the dynamic range and/or sensitivity of the assay may not be adequate to observe the effects of A/T optimization. A/T optimization on a less active background (e.g. L1spa or L1spa_Dbl) could have a pronounced effect on retrotransposition.

This is an excellent point. In response, we have generated the construct smL1_spa3'UTR, which contains the smL1 ORFs and the full L1spa 3'UTR including GRR sequence upstream of the NEO cassette. To our surprise, this construct retrotransposed with similar efficiency to smL1. This data now is included in Figure 2A. We conclude that the negative impact of placing the GRR upstream of the NEO cassette occurs in the context of a natural mouse element, but not a synthetic L1 sequence. We have edited the statement highlighted by Reviewer 2 as follows (pg. 11, Lines 260-264):

"Here we demonstrate that synthetic reduction in mouse L1 ORF adenosine content is not responsible for the previously reported difference in retrotransposition efficiency of smL1 compared to L1spa. We find instead that the elevated activity of smL1 over pTN201/L1spa arises partly from the nonsynonymous ORF1 substitution H159D (46), and to a larger extent from the presence of the 3'UTR GRR sequence upstream of the NEO cassette in the L1spa construct."

We have added the following text to the Results section (Lines 126-135):

"To determine the impact of the GRR in the context of a synthetic L1 element, we generated smL1_spa3'UTR, which contains synthetic smL1 ORF sequences and the full GRR-bearing L1spa 3'UTR. Surprisingly, replacing the GRR into smL1 did not diminish retrotransposition relative to the original smL1 construct (Figure 2A). Taken together, our results indicate that the previously reported improvement in smL1 retrotransposition over L1spa is not attributable to the increased GC content of the smL1 ORFs, but to the combined deleterious effect of the H159 substitution and inclusion of the GRR upstream of the NEO cassette in the pTN201/L1spa construct. While the presence of a histidine residue rather than aspartic acid at ORF1p position 159 diminishes retrotransposition of both L1spa and smL1 (Figure 1C), retrotransposition of the synthetic mouse L1 was not affected by inclusion of the GRR upstream of the NEO cassette. "

We also speculate on the reason for this result in the Discussion (pg. 12-13, Lines 294-322):

"Our results suggest that the molecular mechanism by which deleting or repositioning the L1spa GRR impacts natural mouse L1 retrotransposition efficiency involves modulation of ORF1 protein levels. Relative to a natural mouse L1 construct lacking the GRR (L1spa_Dbl_smBB), placement of the GRR upstream of the NEO cassette (L1spa_Dbl or L1spa_Dbl_smBB_restore_3'UTR) diminishes ORF1p levels 2-fold, and its placement downstream of the NEO cassette, proximal to the 3' end of the L1 transcriptional unit (L1spa_Dbl_smBB_GRRAN), increases ORF1p levels ~1.6-fold (Figure 3C-D, Supplemental Figure 6, Supplemental Figure 7). The position-dependent impact of the GRR on natural mouse L1 ORF1p levels contrasts with our results for L1 RNA levels, which appear to be diminished by the inclusion of the GRR regardless of its placement within the construct (Supplemental Figure 4). ORF1p levels from a synthetic mouse L1 construct lacking the GRR (smL1) are elevated ~15-fold over the congruent natural L1 construct (L1spa_Dbl_smBB), consistent with enhanced protein expression from codon-optimized ORFs, and are modestly reduced by inclusion of the GRR upstream of the NEO cassette (smL1_spa3'UTR)(Figure 3C-D, Supplemental Figure 6). Critically, all the above-described constructs retrotranspose with similar efficiency except for L1spa_Dbl and L1spa_Dbl_smBB_Restore_3'UTR, which retain only ~20% relative retrotransposition efficiency (Figure 1-2, Supplemental Figure 3)."

Of note, the smL1 retrotransposition measurements all have very low variance compared to other constructs. Maybe this reflects increased activity.

The zero variance shown for the construct smL1 in Figure 1C and Figure 2A and L1spa_Dbl_smBB in Figure 2B and Supplemental Figure 3A is a result of setting the smL1 or L1spa_Dbl_smBB colony count to 100% for each biological replicate experiment, and expressing the mean retrotransposition efficiency of other constructs as a percentage of smL1 activity, plus and minus percent standard deviation among biological replicates.

c) The authors should discuss or test the effect of the non-GRR region of the 3'UTR, which contains many changes, is not fully considered in their analysis. Testing variants like L1spa 3'UTR w/ GRR after Neo or L1spa_Dbl_smBB-GRR_before_Neo could provide a more comprehensive understanding of these elements' contributions.

We have generated the construct L1spa_Dbl_SMBB_restore_L1spa_nonGRR, which contains only the first non-GRR portion of the L1spa 3'UTR. This construct, which now is included in Supplemental Figure 3A, retrotransposes with similar efficiency to L1spa_Dbl_smBB, demonstrating that the non-GRR portion of the L1spa 3'UTR does not, by itself, diminish retrotransposition when placed upstream of the NEO cassette. We have included the following in the Results section (Lines 142-146):

"To test whether the non-GRR portion of the L1spa 3'UTR sequence impacts retrotransposition, we generated L1spa_Dbl_smBB_Restore_L1spa_nonGRR (Supplemental Figure 3A). This construct retrotransposed similarly to L1spa_Dbl_smBB, indicating that the non-GRR portion of the L1spa 3'UTR by itself has little impact on retrotransposition efficiency."

d) (line 162) "we replaced the L1spa GRR with the consensus GRR sequence from each of these subfamilies, which contain 9 (G F , A) or 10 (V, F) runs of 3 or more guanine residues (6). In all cases, retrotransposition was reduced to <20% of L1spa_Dbl_smBB (Figure 4A)."

The interpretability of the data in Figure 4 is limited by the lack of a control containing no L1 3'UTR sequence - delta GRR (red) + delta non-GRR-3'UTR sequence (light or dark blue) or a construct with only the GRR and delta non-GRR-3'UTR sequence.

In the former Figure 4, now Supplemental Figure 3A, the construct previously named "L1spa_GRR" contains only the GRR sequence of L1spa, with the non-GRR sequence deleted. For clarity, we have renamed this construct L1spa_Dbl_smBB_Restore_L1spa_GRR. This control is described in manuscript as follows (pg. 6, Lines 146-149):

"In contrast, an L1spa_Dbl construct containing the L1spa GRR and lacking the remaining 3'UTR sequence retrotransposed comparably to L1spa_Dbl, confirming that the L1spa GRR alone is sufficient to impede retrotransposition of a natural mouse L1 (L1spa_Dbl_smBB_Restore_L1spa_GRR, Supplemental Figure 3A)."

2) The authors should clarify impact of these findings on previous/future experiments in the abstract and discussion. Specifically, they should spell out more explicitly that because L1s have been cloned into a vector in which the reporter sequence comes after the GRR of the L1 sequence, activity is depressed. In nature, the GRR is near the end of the transcript, and the change in placement results in this activity depression. They don't completely work out exactly why ORFeus is more active, but do show that the way we clone and measure mouse L1 retrotransposition may underestimate activity.

To clarify our abstract and discussion, we have added the following:

Abstract (pg. 1, Lines 26-28): “Our results prompt a reevaluation of synthetic L1 activity and suggest that the manner in which L1 sequences are cloned into engineered reporter vectors has, in some cases, resulted in an underestimation of native mouse L1 retrotransposition efficiency.”

Discussion (pg. 11, Lines 274-277): “Moreover, our findings emphasize that deleting or reconfiguring portions of the L1 sequence when generating engineered reporter constructs can dramatically impact detected retrotransposition efficiency and complicate interpretation of experimental results.”

Discussion (pg. 13-14, Lines 308-314): “Taken together, our results suggest that the inhibitory effect of the GRR sequence on retrotransposition is position-dependent and may be specific to engineered L1 reporters. Indeed, the possibility that disruption of the L1 3’UTR by an indicator cassette could impact retrotransposition has long been acknowledged (70) and, to speculate, the conservation of a self-attenuating motif is difficult to reconcile with the evolutionary imperative of L1 as a selfish genetic element (20, 22, 71). Positioning the GRR near the 3’ end of the L1 transcriptional unit, where it is found in endogenous L1 elements, does not elevate retrotransposition efficiency more than deleting the GRR entirely, but it does cause a modest increase in ORF1p expression (Figure 3C-D, Supplemental Figure 6, Supplemental Figure 7). Perhaps this positive influence on ORF1p (and speculatively, perhaps ORF2p) expression is more important for endogenous L1 activity *in vivo* than is reflected in an engineered reporter system. The extent to which the deletion of the GRR from the smL1 construct and its derivatives impacts the conclusions drawn from previous *in vitro* and *in vivo* studies using these reporters remains to be evaluated (44, 55-64); at least some transgenic mouse models generated using smL1 based constructs lack the GRR (56, 60, 63, 64). Engineered mouse L1 constructs reconfigured with the GRR downstream of the reporter cassette represent useful tools for investigating the function of this conserved motif, and for studies of mouse L1 biology in general, as high efficiency retrotransposition and structurally normal L1 integrants can be achieved using a construct containing the entire native mouse L1 sequence.”

Minor concerns:

Mention all changes between spa and sm including 3 nt changes interorf spacer and GAGCT deletion just after ORF2 stop (removes SacI site).

The description of the smL1 construct now reads as follows (pg. 4, Lines 95-100):

“In addition, in the smL1 3’UTR nucleotides 2-18, encompassing a SacI restriction site, have been replaced with an 11 nucleotide sequence including an engineered AgeI restriction site, and an engineered PacI site in the inter-ORF spacer sequence introduces three nucleotide changes. The remaining smL1 3’UTR contains 19 nucleotide substitutions and two single nucleotide deletions relative to the L1spa 3’UTR, which arose during synthesis of the smL1 construct (Supplemental Figure 1).”

"This result 184 is consistent with the modest increase in retrotransposition achieved by augmenting L1 expression 185 with a strong heterologous promoter (33), and suggests that L1 mRNA levels are not the rate-limiting 186 determinant of retrotransposition efficiency"

-qualify that this is true when the L1 is highly expressed and might be different with e.g. the native L1 5'UTR promoter.

"yield massively increased mRNA levels relative to native ORF sequences, this 183 elevated expression does not effect a proportionate increase in retrotransposition (1, 42)."

-edit to 'does not "alone" effect'

-related to previous comment - not rate limiting in expression from heterologous promoter, native promoter certainly decreases activity.

Incorporating the above two points, this section now reads as follows (pg. 11, Lines 268-278):

"Thus, while both human and mouse synthetic L1 ORFs yield increased L1 RNA levels relative to native ORF sequences, this elevated expression alone does not effect a proportionate increase in retrotransposition (Figure 1, Figure 2, Supplemental Figure 4)(1, 43). This result is consistent with the modest increase in retrotransposition achieved by augmenting L1 expression with a strong heterologous promoter (19). While regulation of L1 expression is a critical mechanism for limiting endogenous retrotransposition (67), our results suggest that in contexts where L1 expression surpasses a certain threshold, L1 mRNA levels are not the limiting determinant of retrotransposition efficiency. Moreover, our findings emphasize that deleting or reconfiguring portions of the L1 sequence when generating engineered reporter constructs can dramatically impact detected retrotransposition efficiency and complicate interpretation of experimental results."

The data in Figure 3 clearly show that retrotransposed RNAs are being properly processed/polyA'd, but transcript levels could have changed because of RNA polymerase impediment or other causes. The authors could test this or minimally mention that this is a possibility.

We thank Reviewer 3 for this insightful comment. As outlined in our response to Reviewer 2, our qRT-PCR results, shown in Supplemental Figure 4, demonstrate that while inclusion of the GRR within the L1spa transcriptional unit (either upstream or downstream of the NEO cassette) appears to diminish L1 RNA levels, this modulation does not correlate with or explain the observed differences in retrotransposition efficiency among natural mouse L1 constructs (Figure 2).

"We therefore 122 investigated whether formation of a G-quadruplex secondary structure within the GRR diminished 123 retrotransposition via a transcriptional block when placed upstream of the NEO cassette"

-while a G-quadruplex has been biophysically characterized in human L1s, I don't know of similar data for mouse. Rephrase.

The original sentence implying that the mouse GRR is known to form a G-quadruplex structure has been removed in revision.

The discussion would benefit from a more nuanced discussion of the implications of this work to the sequence determinants of human L1 activity. For example, the referenced papers made mutations in some Gs in human L1s (this data is not the most robust due to very low % GFP positive cells in retrotransposition assay) or a short deletion before the reporter. Larger deletion had little to no effect on activity. Also, is there an inference to be made about human from the Figure 4C G1 construct, where addition or deletion of G-rich sequence decreases activity?

This section of the discussion now has been expanded as follows (pg. 13-14, Lines 324-341):

"The GRR sequences from both active and ancient mouse L1 subfamilies diminish natural mouse L1 retrotransposition efficiency when placed upstream of the NEO cassette (Supplemental Figure 3A). Previous reports suggest that the equivalent region of the human L1 3'UTR does not have the same effect on engineered human L1 retrotransposition. The human L1 3'UTR poly-purine tract comprises four runs of 3 to 5 guanine residues across a 34 bp region, which have the demonstrated ability to participate in G-quadruplex secondary structures (22, 68, 69), and in frequently used human L1 reporter constructs this 3'UTR sequence is situated upstream of the NEO cassette (19). In contrast to our results with a natural mouse L1 element, deleting 145 bp of the human 3'UTR including the poly-purine tract in a NEO-based human L1 reporter construct diminished retrotransposition efficiency by ~10% (19). In a subsequent study, deletion of only the 34 bp poly-purine tract diminished retrotransposition of an EGFP-based L1 reporter construct by ~30%, as did mutation of 12 guanine residues to ablate G-quadruplex secondary structure formation; small molecule G-quadruplex stabilization also was shown to very modestly stimulate engineered retrotransposition (69). Future studies are required to determine whether human L1 poly-purine tract presence and position within engineered reporter constructs impact L1 RNA expression and protein expression, and how these potential effects are related to the formation and stabilization of G-quadruplex structures."

Clarify that the presence of the human L1 polyA results from the reporter construction. Is the weak endogenous/mouse L1 polyA also included in the construct?

The endogenouse mouse L1 polyA is not included in the construct. The Figure 1 legend now clarifies that the human L1 polyA signal is "residual from original human retrotransposition indicator constructs (6, 19)".

Why are there 2 polyA signals on the L1-sense strand?

The two polyA signals are the human endogenous polyA left over from the original human L1 retrotransposition indicator constructs, and the SV40 polyadenylation signal from the pCEP4 plasmid backbone.

Data presentation concerns and suggestions:

The graphical depictions of the many construct variants are hard to parse and compare within and between figures. Figure 1B could be a more detail-labeled version of the graphic to define all of the parts, label A/T content in each to highlight key differences in the constructs, which could serve as a reference for all of the shapes, shades, and colors in the figures. Generally, the construct depiction with detailed labeling in figure 3 and 4C is more understandable.

We now have included more detailed labelling in Figure 1B.

The authors could consider including a table with all variables defined for all constructs (see attachment).

This is an excellent suggestion. We now have included Table 1 which summarises all construct characterisitics.

The construct names are also, in many cases, very long and confusing and could be made more interpretable with labeling in figures or name changes (what does smBB stand for?).

We appreciate that the construct names are long and become elaborate as more variations are included. We have respectfully chosen to keep most construct names the same, to ensure consistency between the manuscript, the data supporting each figure, and our own record keeping. We have clarified in the text that smBB stands for "smL1 backbone"—denoting constructs containing a Kozak sequence and the smL1 deletion variant 3'UTR, and additional variations indicated in the individual construct name.

Fig 1C, L1spa_H159D is longer - these need to be aligned.

All constructs in Figure 1C are now aligned.

HSV-TK polyA = black lolli; not defined in figure, only in legend.

HSV-TK polyadenylation signal and SV40 promoter on the antisense NEO cassette now are explicitly described in the figure legend and annotated in Figure 1B.

Why are repeats different shapes? Is there some connection between e.g. triangles across constructs/bars?

Each biological replicate experiment is represented by a different shape.

Figure 3 and 4B were apparently made by a different person from the rest of the figures. It would be good to stylistically normalize the figures.

We have respectfully chosen to retain fundamentally the original figures. Different colour schemes are necessary to illustrate specific components of the reporter constructs relevant to each assay.

-L1spa_Dbl_smBB_GRRAN is the same as L1spa_Dbl_smBB GRR_after_neo, correct? And also contains kozak and DD mutations (not shown in Fig 3A)? Unify naming between Fig2 and Fig3.

Yes, Reviewer 2 also pointed out this inconsistency. We now define "L1spa_Dbl_smBB_GRRAN" to stand for "L1spa Double, smL1 backbone, G-rich region after NEO" in the text, and we use the abbreviation consistently in all figures. Former Figure 3A (now Figure 3B) schematic has been updated to include these details.

In figure 4A, delta is used to denote two different deletions, the GRR deletion and the 3'UTR deletion. Clarify in the legend ("V_GRR, F_GRR, GF_GRR and A_GRR contain 'ONLY' the GRR portion of the 3'UTR") and figure, if possible.

The different 3'UTR deletions now have been clarified in the figure and legend.
