# Supplementary material for: Revisiting the impact of synthetic ORF sequences on engineered LINE-1 retrotransposition
Source: PLoS Genet. 2026 Oct 16;22(10):e1012340. doi: 10.1371/journal.pgen.1012340 (PMC13705589; doi:10.1371/journal.pgen.1012340)
Supplement: Supplementary file 10 [file pgen.1012340.s011.pdf]

Response to Reviewers

Reviewer #1: Review of Chan and Workman et al. entitled, “Revisiting the impact of synthetic ORF sequences on engineered LINE-1 retrotransposition” for PLoS Genetics.

Summary: The authors have satisfactorily addressed my previous comments and, in my opinion, those of the other reviewers in this revised manuscript. The revised manuscript is important, as it forces a reevaluation of some interpretations learned from the use of ‘synthetic’ L1s in retrotransposition assays. It remains clear that ‘synthetic’ L1s may allow more robust expression of L1 mRNA and ORF1p in cultured cells; however, it also is clear that the codon optimization process introduced occult mutations in the ‘synthetic’ mouse L1 sequence and depleted the AU-rich content of L1 RNA.

That being stated, I do have some minor comments for the authors to consider when preparing a revised manuscript; they involve readdressing some overly general discussion points and are noted below. I hope the authors consider briefly addressing these points during the revision process, as it will increase the precision of the manuscript and more fairly acknowledge points made in previous papers.

(1) Discussion, line 275: the sentence “Moreover, our findings…”. The sentence is overly general and vague. Could the authors increase the precision of their statement, perhaps by including an illustrative example(s) of specific things that require reinterpretation (also see below). Clearly, ‘synthetic’ L1s have been useful for studying some aspects of L1 biology. Please consider editing the sentence appropriately.

We thank Reviewer 1 for their feedback on the discussion section. We agree that a more nuanced perspective on studies employing synthetic L1 constructs is warranted. We therefore have removed the clause “and complicate interpretation of experimental results.” from the above sentence and expanded our discussion of previous studies in response to point 2.

(2) Discussion, line 333: the sentence “The extent to which the deletion…”. The authors refer to a list of studies that might require reevaluation. In truth, many of these studies have already acknowledged difference in the activities of ‘natural’ vs. ‘synthetic’ L1s, which, in a way, set the stage for the current study. For example, Alisch et al. 2006 provided experimental genetic evidence (and discussion) to account for differences in the initiation of ORF2p translation in ‘natural’ vs. ‘synthetic’ mouse L1s. Similarly, Richardson et al., 2014 demonstrated that APOBEC3A is less effective at inhibiting the retrotransposition of ‘synthetic’ vs. ‘natural’ mouse L1s; could this be due to fewer A3A editing sites in ‘synthetic’ L1 mRNAs? Finally, the use of ‘synthetic’ L1s, which have

limited sequence identity when compared to 'natural' L1s, likely will limit recombination events with 'natural' L1s either during or after their integration in the genome (e.g., see Gilbert, et al., 2002; Symer et al. 2002; Gilbert et al., 2005). In sum, the authors current statements can be criticized as being overly general.

Increasing the precision of their statements to acknowledge differences observed (and acknowledged) in previous studies would be a welcome addition to the manuscript and would provide specific examples about the benefits of using 'natural' L1s instead of 'synthetic' L1s in studies of L1 biology. Indeed, modifying the discussion, in my opinion, also will highlight the biological importance of the current study.

We thank Reviewer 1 for these thoughtful suggestions, and we agree that the discussion of synthetic and natural L1 reporter use should be more precise. We have revised the final paragraph of the Discussion to emphasise the experimental value of synthetic L1 constructs to previous and future studies, and we also have revised the Abstract and Author Summary to better frame the biological and methodological value of our study.

Abstract (pg 1-2, lines 21-31): "We find that the combined effect of the altered ORF1p amino acid sequence and the GRR 3'UTR deletion, rather than synthetic ORF sequences, accounts for the increase in smL1 retrotransposition efficiency over L1spa. We find that inclusion of the GRR influences natural mouse L1 retrotransposition in a position-dependent manner that does not involve premature polyadenylation of L1 mRNA transcripts or alteration of L1 insertion structures. Instead, we present evidence that the position-dependent effect of the GRR on natural mouse retrotransposition occurs through modulation of L1 ORF1p abundance. In contrast, smL1 retrotransposition is refractory to the inhibitory effect of the GRR, likely owing to highly efficient protein expression from synthetic ORF sequences. Taken together, our results prompt further investigation of the relationship between the GRR sequence and L1 protein expression and demonstrate that the arrangement of L1 nucleotide sequences within reporter constructs can influence L1 expression and engineered retrotransposition efficiency."

Author Summary (pg. 2, lines 45-47): "Our study reveals a potential role for the GRR sequence in L1 protein expression and will inform the ongoing use of synthetic and natural mouse L1 reporter constructs in vivo and in vitro."

Discussion (pg.15-17, lines 343-387): Due to their high retrotransposition efficiency and abundant expression of L1 RNA and protein, synthetic L1 constructs have tremendous utility for investigating diverse facets of L1 biology (1-15). Synthetic L1 constructs can be particularly powerful experimental tools when used alongside natural L1 reporters, and numerous studies have leveraged this experimental paradigm while acknowledging the relevant differences between synthetic and natural L1 retrotransposition. Comparison

of synthetic and natural L1 reporters has provided insight regarding the L1 sequence dependence of host restriction factors that modulate L1 activity. For example, the human silencing hub (HUSH) complex initially was reported to silence natural but not synthetic engineered L1 insertions (15), and subsequent work demonstrated that the HUSH complex effects epigenetic repression of intronless transposable element sequences in a manner dependent on elevated sense strand adenosine content (16). In addition, the cytidine deaminase APOBEC3A (A3A), an antiviral and anti-transposable element restriction factor (17, 18), inhibits retrotransposition of smL1 less severely than natural LINE elements from human, mouse and zebrafish (9). This difference suggests that while A3A inhibition of L1 is not strictly sequence-dependent, the altered sequence composition of the smL1 cDNA may be depleted for preferred A3A editing substrates and therefore refractory to A3A-mediated deamination and subsequent degradation. In addition, it is likely that the abundance of functional L1 RNPs generated by synthetic elements can overcome the effects of cellular restriction factors upon retrotransposition and also can compensate for cis-acting mutations within the L1 coding sequence. Indeed, in the context of a natural mouse L1, the H159 ORF1p mutation diminishes retrotransposition efficiency ~3 to 5-fold (Figure 1C, compare L1spa or L1spa_G53D to L1spa_Dbl; and Figure 2B, compare L1spa_smBB to L1spa_Dbl_smBB), while the same mutation in smL1 diminishes retrotransposition efficiency less than 2-fold (Figure 1C, compare smL1_D159H or smL1_Dbl to smL1). Similarly, a study demonstrating unconventional L1 ORF2p translation initiation found that both natural and synthetic mouse L1 constructs with mutated ORF2p AUG initiation codons retain retrotransposition competence. However, while such mutations diminished natural mouse L1 retrotransposition by ~70-80%, synthetic mouse L1 retrotransposition was unimpaired, likely due to the elevated production of functional L1 RNPs by the synthetic element (1).

Synthetic L1 constructs offer unique experimental advantages for future studies, as long-read sequencing is increasingly applied to map and characterize integrants arising from ectopically expressed L1 reporter constructs (19-21). Synthetic L1 expression vectors could be used to generate reporterless insertions readily distinguishable from genomic L1s and amenable to high-depth sequencing using Cas9 targeted enrichment or adaptive sampling approaches (22-24). Furthermore, L1 cDNAs from synthetic L1 constructs are less likely than those from natural L1 constructs to recombine with existing L1s in the genome during or after integration. Synthetic and natural L1 constructs therefore could be used in tandem to gain insight about role of sequence homology in L1-mediated structural rearrangements that occur during TPRT (25-27), including large-scale chromosomal aberrations recently observed in human tumor genomes (28, 29).

The findings presented here may benefit future studies by ensuring that when synthetic and natural mouse L1 constructs used together they contain complete noncoding L1

sequences, encode identical L1 proteins, and are structured the same way regarding placement of the retrotransposition indicator cassette. Furthermore, our work demonstrates that it is possible to generate natural mouse L1 constructs that encompass the entire L1 sequence and are capable of high efficiency retrotransposition comparable to smL1 (e.g., L1spa_Dbl_smBB_GRRAN). Such constructs likely will be useful in situations where it is important that engineered L1 RNA and protein expression levels are similar to expression levels from an endogenous element, and where retention of the complete native L1 nucleotide sequence is directly relevant to the biological question under investigation."

Reviewer #2: The authors have done an excellent job addressing my major concerns. They appropriately moderated several causal claims and added a strong set of constructs and controls that disentangle the contributions of the ORF1 amino acid substitutions, the GRR, the non-GRR portion of the 3'UTR, and the position of these sequences relative to the reporter cassette. In particular, the reciprocal mutations in smL1, restoration of the native 3'UTR to smL1, and testing of GRR deletion or repositioning in the original L1spa background substantially strengthen the central conclusions. The new qRT-PCR and ORF1p Western blot experiments also provide useful mechanistic insight while appropriately acknowledging the questions that remain. Finally, the revised figures, construct summary table, and expanded discussion greatly improve the clarity and interpretation of the study. This is an important paper because it resolves a longstanding discrepancy regarding the effects of synthetic mouse L1 sequences and demonstrates that reporter architecture can substantially influence measured retrotransposition activity. These findings have broad implications for the interpretation of prior studies and for the design of future mouse L1 assays. I am satisfied that my concerns have been addressed and support publication. ~Rick McLaughlin

We thank Reviewer 2 for their supportive comments.

Reviewer #3: Summary:

Chan et al. evaluated the retrotransposition rates between endogenous mouse L1 sequence (L1spa) versus a previously published synthetic mouse L1 sequence (smL1) with reduced open reading frame (ORF) adenosine content, which are both widely used experimental tools in the L1 biology field. Through sequencing of the smL1 construct, the authors find that the smL1 sequence contains additional nonsynonymous coding mutations in ORF1 and significant alterations to noncoding sequences in addition to the

intended reduced ORF adenosine content. Due to these unanticipated discrepancies and, therefore, the inability to make 1:1 comparisons between smL1 and L1spa, the authors sought to determine whether any of these additional changes in smL1 were responsible for the observed high rates of retrotransposition for smL1. Through well-controlled and thorough experiments, Chan et al. show compelling data that the H159D mutation in ORF1p and loss of the GRR in the 3'UTR account for the high rates of retrotransposition of synthetic smL1 sequence rather than reduced adenosine content originally proposed. While the mechanistic understanding of these alterations remains unclear, the authors propose a model where, in a natural L1 element, loss of the GRR increases ORF1p protein levels and this in turn increases L1 retrotransposition. The novelty and impact of these findings lie in the transparency about the discovered confounders in these widely used L1 retrotransposition constructs. Additionally, these findings nucleate future research into noncoding parts of the L1 sequence and how they influence L1 retrotransposition. This manuscript is suitable for publication with PLoS Genetics upon completion the following comments.

Comments:

1. The constructs used here are derived from mouse-specific L1 elements, yet all the experiments in this manuscript are performed in HeLa cells. Thus, there may be species-specific regulations due to the coevolution of L1 and its host that are unaccounted for in the authors' experiments. Validating key findings (e.g. Figure 2) regarding retrotransposition frequency, RNA levels, and ORF1p protein levels in mouse cells would strengthen the author's findings and conclusions.

This is a valid point. We agree that mouse-specific L1 regulatory mechanisms could impact the retrotransposition efficiency of our reporter constructs, and that these potential impacts are not reflected in human HeLa cell assays. However, there are also advantages to using a human cell line, as measurements of mouse L1 RNA and L1 ORF1p levels from transfected expression vectors are not confounded by the presence of endogenous mouse L1 RNA and protein. We now explicitly acknowledge these points in the Discussion (page 15, line 334-342):

"Moreover, all experiments in this study were carried out in human HeLa cells. We chose this experimental system to remain consistent with the original report describing the smL1 construct (30), and so that detection of ectopically expressed mouse L1 RNA and ORF1p would not be confounded by endogenous L1 expression. However, it is possible that effect of the GRR on mouse L1 retrotransposition is influenced mouse-specific L1 regulatory mechanisms absent from human cells. As a cancer cell line, HeLa cells also do not reflect *in vivo* niches for heritable L1 retrotransposition—pluripotent embryonic cells and cells of the germ lineage (21, 31-34). Future

experiments using mouse cell lines may reveal species- and cell type-specific relationships between GRR presence, ORF1p levels, and retrotransposition efficiency."

2. The proposed model that ORF1p levels are affected by the GRR are weakly supported by the data presented (Figure 3C, D). Performing and quantifying western blots probing for ORF1p alongside the data in Supplementary Figure 3A, C to show a decrease in ORF1p levels corresponding with GRRs from ancient L1s (Sup Fig 3A) as well as while the GRR length increases (Sup Fig 3C) would greatly substantiate this hypothesis.

We respectfully disagree that the data indicating changes in ORF1p levels (Figure 3C, D) are "weakly" supported. As stated in the text and supported by the inclusion of all replicate blots in Supplemental Figure 6, when performing ORF1p quantitation each construct was used in three independent transfections (biological replicates), and cell lysate from each biological replicate was used to perform two technical replicate blots. Moreover, the key result that GRR presence and placement in a natural mouse L1 construct impacts ORF1p levels is recapitulated in an entirely separate set of transfections and blots (Supplemental Figure 7), performed with the same experimental rigor. We therefore have not performed Western blots using constructs bearing GRR sequences from other mouse L1 subfamilies or progressively deleted GRR sequences (comprising 13 additional constructs plus controls), but we have amended the Discussion to include the following statement (page 13, lines 291-295):

"We speculate that the constructs containing various mouse L1 subfamily GRR sequences upstream of the NEO cassette (Supplemental Figure 3A) would likewise show diminished ORF1p levels, and that among our deletion series constructs (Supplemental Figure 3B) ORF1p levels would increase incrementally with progressive removal of GRR sequence."

Minor comments:

1. The naming schemes of the many thoughtfully made constructs make the manuscript difficult to read. Editing these to a short-hand naming system that is easier and accessible for all readers would help immensely. For example, using smL1-3'UTR instead of smBB would indicate to the readers the changes being made.

We agree that the naming schemes are cumbersome, but they reflect the iterative generation of modified constructs over the course of the project and in response to reviewer requests. For the sake of consistency between manuscript versions and our own record keeping, we respectfully choose to retain the original construct names.

2. While the retrotransposition data is very clear and compelling in the main Figures and supplementary Figures, they lack statistical tests. Please add where appropriate accounting for multiple comparisons.

We now have performed ANOVA with Tukey's multiple comparisons test for all retrotransposition assay data. Throughout the manuscript, we have updated the figures and text with p-values for comparisons relevant to the conclusions drawn.

3. Figure 3C shows two bands in the ORF1p immunoblot. Please indicate in the main figure which band you consider to be ORF1p and are quantifying in Figure 3D. The blot would benefit from including a positive and negative control samples.

The figure and legend now have been updated with an arrow indicating the ~42kd ORF1p band that has been quantified.

4. The constructs with GRR after NEO are referred to as "GRANN" in Figure 2 but "GRR_after_NEO" in Figure 3. Please adjust for consistency.

This change has been made.

1. Alisch RS, Garcia-Perez JL, Muotri AR, Gage FH, Moran JV. Unconventional translation of mammalian LINE-1 retrotransposons. Genes Dev. 2006;20(2):210-24.
2. An W, Han JS, Schrum CM, Maitra A, Koentgen F, Boeke JD. Conditional activation of a single-copy L1 transgene in mice by Cre. Genesis. 2008;46(7):373-83.
3. An W, Han JS, Wheelan SJ, Davis ES, Coombes CE, Ye P, et al. Active retrotransposition by a synthetic L1 element in mice. Proc Natl Acad Sci U S A. 2006;103(49):18662-7.
4. Grandi FC, Rosser JM, An W. LINE-1-derived poly(A) microsatellites undergo rapid shortening and create somatic and germline mosaicism in mice. Mol Biol Evol. 2013;30(3):503-12.
5. Kannan M, Li J, Fritz SE, Husarek KE, Sanford JC, Sullivan TL, et al. Dynamic silencing of somatic L1 retrotransposon insertions reflects the developmental and cellular contexts of their genomic integration. Mobile DNA. 2017;8:8.
6. Monot C, Kuciak M, Viollet S, Mir AA, Gabus C, Darlix JL, et al. The specificity and flexibility of l1 reverse transcription priming at imperfect T-tracts. PLoS Genet. 2013;9(5):e1003499.
7. Newkirk SJ, Lee S, Grandi FC, Gaysinskaya V, Rosser JM, Vanden Berg N, et al. Intact piRNA pathway prevents L1 mobilization in male meiosis. Proc Natl Acad Sci U S A. 2017;114(28):E5635-E44.
8. O'Donnell KA, An W, Schrum CT, Wheelan SJ, Boeke JD. Controlled insertional mutagenesis using a LINE-1 (ORFeus) gene-trap mouse model. Proc Natl Acad Sci U S A. 2013;110(29):E2706-13.

9. Richardson SR, Narvaiza I, Planegger RA, Weitzman MD, Moran JV. APOBEC3A deaminates transiently exposed single-strand DNA during LINE-1 retrotransposition. eLife. 2014;2014(3).
10. Wagstaff BJ, Barnerssoi M, Roy-Engel AM. Evolutionary conservation of the functional modularity of primate and murine LINE-1 elements. PLoS One. 2011;6(5):e19672.
11. Xie Y, Mates L, Ivics Z, Izsvak Z, Martin SL, An W. Cell division promotes efficient retrotransposition in a stable L1 reporter cell line. Mobile DNA. 2013;4(1):10.
12. Mita P, Wudzinska A, Sun X, Andrade J, Nayak S, Kahler DJ, et al. LINE-1 protein localization and functional dynamics during the cell cycle. Elife. 2018;7.
13. Taylor MS, Altukhov I, Molloy KR, Mita P, Jiang H, Adney EM, et al. Dissection of affinity captured LINE-1 macromolecular complexes. Elife. 2018;7.
14. Taylor MS, LaCava J, Mita P, Molloy KR, Huang CR, Li D, et al. Affinity proteomics reveals human host factors implicated in discrete stages of LINE-1 retrotransposition. Cell. 2013;155(5):1034-48.
15. Liu N, Lee CH, Swigut T, Grow E, Gu B, Bassik MC, et al. Selective silencing of euchromatic L1s revealed by genome-wide screens for L1 regulators. Nature. 2018;553(7687):228-32.
16. Seczynska M, Bloor S, Cuesta SM, Lehner PJ. Genome surveillance by HUSH-mediated silencing of intronless mobile elements. Nature. 2022;601(7893):440-5.
17. Bogerd HP, Wiegand HL, Hulme AE, Garcia-Perez JL, O'Shea KS, Moran JV, et al. Cellular inhibitors of long interspersed element 1 and Alu retrotransposition. Proc Natl Acad Sci U S A. 2006;103(23):8780-5.
18. Chen H, Lilley CE, Yu Q, Lee DV, Chou J, Narvaiza I, et al. APOBEC3A is a potent inhibitor of adeno-associated virus and retrotransposons. Curr Biol. 2006;16(5):480-5.
19. Gerdes P, Lim SM, Ewing AD, Larcombe MR, Chan D, Sanchez-Luque FJ, et al. Retrotransposon instability dominates the acquired mutation landscape of mouse induced pluripotent stem cells. Nat Commun. 2022;13(1):7470.
20. Mendez-Dorantes C, Kalinowski JC, Law CT, Schofield P, Burn A, Burns KH. L1 insertion intermediates recombine with one another or with DNA breaks to form genome rearrangements. bioRxiv. 2025.
21. de Los Rios Barreda J, Ferreiro ME, Jansz N, Bell CC, Botto JM, Nguyen TV, et al. X-chromosome inactivation draws L1 mutagenesis to the human X chromosome. Science. 2026;393(6810):eadz8081.
22. McDonald TL, Zhou W, Castro CP, Mumm C, Switzenberg JA, Mills RE, et al. Cas9 targeted enrichment of mobile elements using nanopore sequencing. Nat Commun. 2021;12(1):3586.
23. Sarkar A, Lanciano S, Cristofari G. Targeted Nanopore Resequencing and Methylation Analysis of LINE-1 Retrotransposons. Methods Mol Biol. 2023;2607:173-98.
24. Loose M, Malla S, Stout M. Real-time selective sequencing using nanopore technology. Nat Methods. 2016;13(9):751-4.
25. Gilbert N, Lutz S, Morrish TA, Moran JV. Multiple fates of L1 retrotransposition intermediates in cultured human cells. Mol Cell Biol. 2005;25(17):7780-95.
26. Gilbert N, Lutz-Prigge S, Moran JV. Genomic deletions created upon LINE-1 retrotransposition. Cell. 2002;110(3):315-25.

27. Symer DE, Connelly C, Szak ST, Caputo EM, Cost GJ, Parmigiani G, et al. Human l1 retrotransposition is associated with genetic instability in vivo. Cell. 2002;110(3):327-38.
28. Zumalave S, Santamarina M, N PE, Zamora J, Garcia-Souto D, Temes J, et al. Concurrent L1 retrotransposition events promote reciprocal translocations in human tumorigenesis. Science. 2026;392(6793):eaee4513.
29. Rodriguez-Martin B, Alvarez EG, Baez-Ortega A, Zamora J, Supek F, Demeulemeester J, et al. Pan-cancer analysis of whole genomes identifies driver rearrangements promoted by LINE-1 retrotransposition. Nat Genet. 2020;52(3):306-19.
30. Han JS, Boeke JD. A highly active synthetic mammalian retrotransposon. Nature. 2004;429(6989):314-8.
31. Garcia-Perez JL, Marchetto MC, Muotri AR, Coufal NG, Gage FH, O'Shea KS, et al. LINE-1 retrotransposition in human embryonic stem cells. Hum Mol Genet. 2007;16(13):1569-77.
32. Kano H, Godoy I, Courtney C, Vetter MR, Gerton GL, Ostertag EM, et al. L1 retrotransposition occurs mainly in embryogenesis and creates somatic mosaicism. Genes Dev. 2009;23(11):1303-12.
33. Richardson SR, Faulkner GJ. Heritable L1 Retrotransposition Events During Development: Understanding Their Origins: Examination of heritable, endogenous L1 retrotransposition in mice opens up exciting new questions and research directions. Bioessays. 2018;40(6):e1700189.
34. Richardson SR, Gerdes P, Gerhardt DJ, Sanchez-Luque FJ, Bodea GO, Munoz-Lopez M, et al. Heritable L1 retrotransposition in the mouse primordial germline and early embryo. Genome Res. 2017;27(8):1395-405.
