# Supplementary material for: Revisiting the impact of synthetic ORF sequences on engineered LINE-1 retrotransposition
Source: PLoS Genet. 2026 Oct 16;22(10):e1012340. doi: 10.1371/journal.pgen.1012340 (PMC13705589; doi:10.1371/journal.pgen.1012340)
Supplement: Peer review history

# Author comment: Author response to Decision Letter 0

**DOI:** 10.1371/journal.pgen.1012340.r001

8 Jun 2026

Attachment Submitted filename: Chan_Response to reviewers.pdf

# Peer review: Decision Letter 0

**DOI:** 10.1371/journal.pgen.1012340.r002

**Authors:** Paula Cohen, Michael Metzger

7 Aug 2026

PGENETICS-D-26-00598

Revisiting the impact of synthetic ORF sequences on engineered LINE-1 retrotransposition

PLOS Genetics

Dear Dr. Richardson,

Thank you for submitting your manuscript to PLOS Genetics. After careful consideration, we feel that it has merit but does not fully meet PLOS Genetics's publication criteria as it currently stands. Therefore, we invite you to submit a revised version of the manuscript that addresses the points raised during the review process.

Please submit your revised manuscript within by Sep 06 2026 11:59PM. If you will need more time than this to complete your revisions, please reply to this message or contact the journal office at plosgenetics@plos.org. When you're ready to submit your revision, log on to https://www.editorialmanager.com/pgenetics/ and select the 'Submissions Needing Revision' folder to locate your manuscript file.

Please include the following items when submitting your revised manuscript:

* A letter that responds to each point raised by the editor and reviewer(s). You should upload this letter as a separate file labeled 'Response to Reviewers'. This file does not need to include responses to formatting updates and technical items listed in the 'Journal Requirements' section below.

* A marked-up copy of your manuscript that highlights changes made to the original version. You should upload this as a separate file labeled 'Revised Manuscript with Track Changes'.

* An unmarked version of your revised paper without tracked changes. You should upload this as a separate file labeled 'Manuscript'.

If you would like to make changes to your financial disclosure, competing interests statement, or data availability statement, please make these updates within the submission form at the time of resubmission. Guidelines for resubmitting your figure files are available below the reviewer comments at the end of this letter.

As the corresponding author, your ORCID iD is verified in the submission system and will appear in the published article. PLOS supports the use of ORCID, and we encourage all coauthors to register for an ORCID iD and use it as well. Please encourage your coauthors to verify their ORCID iD within the submission system before final acceptance, as unverified ORCID iDs will not appear in the published article. Only the individual author can complete the verification step; PLOS staff cannot verify ORCID iDs on behalf of authors.

We look forward to receiving your revised manuscript.

Kind regards,

Michael J Metzger, Ph.D.

Academic Editor

PLOS Genetics

Paula Cohen

Section Editor

PLOS Genetics

Aimée Dudley

Editor-in-Chief

PLOS Genetics

Anne Goriely

Editor-in-Chief

PLOS Genetics

**Additional Editor Comments (if provided):**

The manuscript has been reviewed by three reviewers (two who had reviewed it previously and one new reviewer). Both previous reviewers praised the work and state that the authors have answered all major concerns, although there were some minor concerns that the authors can consider. The new reviewer also had several concerns. This manuscript needs some minor revision at this point. Please consider the concerns by all reviewers. In the case of the two major concerns requesting significant additional data (i.e. repeating the work in murine cells and repeating westerns shown in Fig 3 and S6 with additional ancestral L1 elements), please include new data or include some text acknowedging the limitation of the conclusions given the absense of this data in this manuscript.

**Journal Requirements:**

If the reviewer comments include a recommendation to cite specific previously published works, please review and evaluate these publications to determine whether they are relevant and should be cited. There is no requirement to cite these works unless the editor has indicated otherwise.

1) We ask that a manuscript source file is provided at Revision. Please upload your manuscript file as a .doc, .docx, .rtf or .tex. If you are providing a .tex file, please upload it under the item type u2018LaTeX Source Fileu2019 and leave your .pdf version as the item type u2018Manuscriptu2019.

2) Please provide an Author Summary. This should appear in your manuscript between the Abstract (if applicable) and the Introduction, and should be 150-200 words long. The aim should be to make your findings accessible to a wide audience that includes both scientists and non-scientists. Sample summaries can be found on our website under Submission Guidelines:

https://journals.plos.org/plosgenetics/s/submission-guidelines#loc-parts-of-a-submission

3) We noticed that you used the phrase 'data not shown' in the manuscript. We do not allow these references, as the PLOS data access policy requires that all data be either published with the manuscript or made available in a publicly accessible database. Please amend the supplementary material to include the referenced data or remove the references.

4) Please upload all main figures as separate Figure files in .tif or .eps format. For more information about how to convert and format your figure files please see our guidelines:

https://journals.plos.org/plosgenetics/s/figures

5) Please upload a copy of Figure Fig 1-3 which you refer to in your text on pages 3, and 5 and 8. Or, if the figure is no longer to be included as part of the submission please remove all reference to it within the text.

6) We have noticed that you have uploaded Supporting Information files, but you have not included a list of legends. Please add a full list of legends for your Supporting Information files after the references list.

7) Your current Financial Disclosure states, "This study was funded by the Australian Medical Research Future Fund (MRFF; https://www.health.gov.au/our-work/mrff)(MRF2043804 to S.R.R. and S.W.), the Australian National Health and Medical Research Council (NHMRC; https://www.nhmrc.gov.au)(GNT1125645, GNT1138795, GNT1173711 and GNT2033366 to G.J.F.; GNT1173476 to S.R.R.), The Australian Research Council (ARC; arc.gov.au) (DP210100614 to S.R.R; DP200102919 to G.J.F. and S.R.R.), a CSL (csl.com) Centenary Fellowship to G.J.F., an Advance Queensland (advance.qld.gov.au) Women’s Academic Fund Maternity Funding award to S.R.R., the Mater Research (materresearch.org.au) Strategic Grant for Outstanding Women to S.R.R, the Mater Foundation (materfoundation.org.au), an Australian Government (education.gov.au) Research Training Program Scholarship to P.G. and a Mater Research Frank Clair Scholarship to P.G. The funders did not play any role in the study design, data collection and analysis, decision to publish, or preparation of the manuscript.". However, your funding information on the submission form indicates "order of grants" mismatches. Please indicate by return email the full and correct funding information for your study and confirm the order in which funding contributions should appear. Please be sure to indicate whether the funders played any role in the study design, data collection and analysis, decision to publish, or preparation of the manuscript.

8) Please send a completed 'Competing Interests' statement, including any COIs declared by your co-authors. If you have no competing interests to declare, please state "The authors have declared that no competing interests exist". Otherwise please declare all competing interests beginning with the statement "I have read the journal's policy and the authors of this manuscript have the following competing interests:"

**Reviewers' comments:**

Reviewer's Responses to Questions

**Comments to the Authors:**

**Please note here if the review is uploaded as an attachment.**

Reviewer #1: Review of Chan and Workman et al. entitled, “Revisiting the impact of synthetic ORF sequences on engineered LINE-1 retrotransposition” for PLoS Genetics.

Summary

The authors have satisfactorily addressed my previous comments and, in my opinion, those of the other reviewers in this revised manuscript. The revised manuscript is important, as it forces a reevaluation of some interpretations learned from the use of ‘synthetic’ L1s in retrotransposition assays. It remains clear that ‘synthetic’ L1s may allow more robust expression of L1 mRNA and ORF1p in cultured cells; however, it also is clear that the codon optimization process introduced occult mutations in the ‘synthetic’ mouse L1 sequence and depleted the AU-rich content of L1 RNA.

That being stated, I do have some minor comments for the authors to consider when preparing a revised manuscript; they involve readdressing some overly general discussion points and are noted below. I hope the authors consider briefly addressing these points during the revision process, as it will increase the precision of the manuscript and more fairly acknowledge points made in previous papers.

(1) Discussion, line 275: the sentence “Moreover, our findings…”. The sentence is overly general and vague. Could the authors increase the precision of their statement, perhaps by including an illustrative example(s) of specific things that require reinterpretation (also see below). Clearly, ‘synthetic’ L1s have been useful for studying some aspects of L1 biology. Please consider editing the sentence appropriately.

(2) Discussion, line 333: the sentence “The extent to which the deletion…”. The authors refer to a list of studies that might require reevaluation. In truth, many of these studies have already acknowledged difference in the activities of ‘natural’ vs. ‘synthetic’ L1s, which, in a way, set the stage for the current study. For example, Alisch et al. 2006 provided experimental genetic evidence (and discussion) to account for differences in the initiation of ORF2p translation in ‘natural’ vs. ‘synthetic’ mouse L1s. Similarly, Richardson et al., 2014 demonstrated that APOBEC3A is less effective at inhibiting the retrotransposition of ‘synthetic’ vs. ‘natural’ mouse L1s; could this be due to fewer A3A editing sites in ‘synthetic’ L1 mRNAs? Finally, the use of ‘synthetic’ L1s, which have limited sequence identity when compared to ‘natural’ L1s, likely will limit recombination events with ‘natural’ L1s either during or after their integration in the genome (e.g., see Gilbert, et al., 2002; Symer et al. 2002; Gilbert et al., 2005). In sum, the authors current statements can be criticized as being overly general.

Increasing the precision of their statements to acknowledge differences observed (and acknowledged) in previous studies would be a welcome addition to the manuscript and would provide specific examples about the benefits of using ‘natural’ L1s instead of ‘synthetic’ L1s in studies of L1 biology. Indeed, modifying the discussion, in my opinion, also will highlight the biological importance of the current study.

I commend the authors on conducting a rigorous and thorough analysis.

Reviewer #2: The authors have done an excellent job addressing my major concerns. They appropriately moderated several causal claims and added a strong set of constructs and controls that disentangle the contributions of the ORF1 amino acid substitutions, the GRR, the non-GRR portion of the 3′UTR, and the position of these sequences relative to the reporter cassette. In particular, the reciprocal mutations in smL1, restoration of the native 3′UTR to smL1, and testing of GRR deletion or repositioning in the original L1spa background substantially strengthen the central conclusions. The new qRT-PCR and ORF1p Western blot experiments also provide useful mechanistic insight while appropriately acknowledging the questions that remain. Finally, the revised figures, construct summary table, and expanded discussion greatly improve the clarity and interpretation of the study. This is an important paper because it resolves a longstanding discrepancy regarding the effects of synthetic mouse L1 sequences and demonstrates that reporter architecture can substantially influence measured retrotransposition activity. These findings have broad implications for the interpretation of prior studies and for the design of future mouse L1 assays. I am satisfied that my concerns have been addressed and support publication. ~Rick McLaughlin

Reviewer #3: Summary:

Chan et al. evaluated the retrotransposition rates between endogenous mouse L1 sequence (L1spa) versus a previously published synthetic mouse L1 sequence (smL1) with reduced open reading frame (ORF) adenosine content, which are both widely used experimental tools in the L1 biology field. Through sequencing of the smL1 construct, the authors find that the smL1 sequence contains additional nonsynonymous coding mutations in ORF1 and significant alterations to noncoding sequences in addition to the intended reduced ORF adenosine content. Due to these unanticipated discrepancies and, therefore, the inability to make 1:1 comparisons between smL1 and L1spa, the authors sought to determine whether any of these additional changes in smL1 were responsible for the observed high rates of retrotransposition for smL1. Through well-controlled and thorough experiments, Chan et al. show compelling data that the H159D mutation in ORF1p and loss of the GRR in the 3’UTR account for the high rates of retrotransposition of synthetic smL1 sequence rather than reduced adenosine content originally proposed. While the mechanistic understanding of these alterations remains unclear, the authors propose a model where, in a natural L1 element, loss of the GRR increases ORF1p protein levels and this in turn increases L1 retrotransposition. The novelty and impact of these findings lie in the transparency about the discovered confounders in these widely used L1 retrotransposition constructs. Additionally, these findings nucleate future research into noncoding parts of the L1 sequence and how they influence L1 retrotransposition. This manuscript is suitable for publication with PLoS Genetics upon completion the following comments.

Comments:

1. The constructs used here are derived from mouse-specific L1 elements, yet all the experiments in this manuscript are performed in HeLa cells. Thus, there may be species-specific regulations due to the coevolution of L1 and its host that are unaccounted for in the authors’ experiments. Validating key findings (e.g. Figure 2) regarding retrotransposition frequency, RNA levels, and ORF1p protein levels in mouse cells would strengthen the author’s findings and conclusions.

2. The proposed model that ORF1p levels are affected by the GRR are weakly supported by the data presented (Figure 3C, D). Performing and quantifying western blots probing for ORF1p alongside the data in Supplementary Figure 3A, C to show a decrease in ORF1p levels corresponding with GRRs from ancient L1s (Sup Fig 3A) as well as while the GRR length increases (Sup Fig 3C) would greatly substantiate this hypothesis.

Minor comments:

1. The naming schemes of the many thoughtfully made constructs make the manuscript difficult to read. Editing these to a short-hand naming system that is easier and accessible for all readers would help immensely. For example, using smL1-3’UTR instead of smBB would indicate to the readers the changes being made.

2. While the retrotransposition data is very clear and compelling in the main Figures and supplementary Figures, they lack statistical tests. Please add where appropriate accounting for multiple comparisons.

3. Figure 3C shows two bands in the ORF1p immunoblot. Please indicate in the main figure which band you consider to be ORF1p and are quantifying in Figure 3D. The blot would benefit from including a positive and negative control samples.

4. The constructs with GRR after NEO are referred to as “GRANN” in Figure 2 but “GRR_after_NEO” in Figure 3. Please adjust for consistency.

**********

**Have all data underlying the figures and results presented in the manuscript been provided?**

Large-scale datasets should be made available via a public repository as described in the *PLOS Genetics*  data availability policy, and numerical data that underlies graphs or summary statistics should be provided in spreadsheet form as supporting information.

Reviewer #1: Yes

Reviewer #2: Yes

Reviewer #3: Yes

**********

PLOS authors have the option to publish the peer review history of their article (what does this mean?). If published, this will include your full peer review and any attached files.

If you choose “no”, your identity will remain anonymous but your review may still be made public.

**Do you want your identity to be public for this peer review?**  For information about this choice, including consent withdrawal, please see our Privacy Policy.

Reviewer #1: No

Reviewer #2: **Yes:**  Rick McLaughlin

Reviewer #3: No

[NOTE: If reviewer comments were submitted as an attachment file, they will be attached to this email and accessible via the submission site. Please log into your account, locate the manuscript record, and check for the action link "View Attachments". If this link does not appear, there are no attachment files.]

**Figure resubmission:**

**Reproducibility:**

To enhance the reproducibility of your results, we recommend that authors deposit laboratory protocols in protocols.io, where a protocol can be assigned its own identifier (DOI) such that it can be cited independently in the future. Additionally, PLOS ONE offers an option to publish peer-reviewed clinical study protocols. Read more information on sharing protocols at https://plos.org/protocols?utm_medium=editorial-email&utm_source=authorletters&utm_campaign=protocols

# Author comment: Author response to Decision Letter 1

**DOI:** 10.1371/journal.pgen.1012340.r003

15 Sep 2026

Attachment Submitted filename: Response to reviewers_14-9-2026.pdf

# Editor report: Decision Letter 1

**DOI:** 10.1371/journal.pgen.1012340.r004

**Authors:** Paula Cohen, Michael Metzger

29 Sep 2026

Dear Dr Richardson,

We are pleased to inform you that your manuscript entitled "Revisiting the impact of synthetic ORF sequences on engineered LINE-1 retrotransposition" has been editorially accepted for publication in PLOS Genetics. Congratulations!

Before your submission can be formally accepted and sent to production you will need to complete our formatting changes, which you will receive in a follow up email. Please be aware that it may take several days for you to receive this email; during this time no action is required by you. Please note: the accept date on your published article will reflect the date of this provisional acceptance, but your manuscript will not be scheduled for publication until the required changes have been made.

Once your paper is formally accepted, an uncorrected proof of your manuscript will be published online ahead of the final version, unless you’ve already opted out via the online submission form. If, for any reason, you do not want an earlier version of your manuscript published online or are unsure if you have already indicated as such, please let the journal staff know immediately at plosgenetics@plos.org.

In the meantime, please log into Editorial Manager at https://www.editorialmanager.com/pgenetics/, click the "Update My Information" link at the top of the page, and update your user information to ensure an efficient production and billing process. Note that PLOS requires an ORCID iD for all corresponding authors. Therefore, please ensure that you have an ORCID iD and that it is validated in Editorial Manager. To do this, go to ‘Update my Information’ (in the upper left-hand corner of the main menu), and click on the Fetch/Validate link next to the ORCID field.  This will take you to the ORCID site and allow you to create a new iD or authenticate a pre-existing iD in Editorial Manager.

If you have a press-related query, or would like to know about making your underlying data available (as you will be aware, this is required for publication), please see the end of this email. If your institution or institutions have a press office, please notify them about your upcoming article at this point, to enable them to help maximise its impact. Inform journal staff as soon as possible if you are preparing a press release for your article and need a publication date.

Thank you again for supporting open-access publishing; we are looking forward to publishing your work in PLOS Genetics!

Yours sincerely,

Michael J Metzger, Ph.D.

Academic Editor

PLOS Genetics

Paula Cohen

Section Editor

PLOS Genetics

Aimée Dudley

Editor-in-Chief

PLOS Genetics

Anne Goriely

Editor-in-Chief

PLOS Genetics

www.plosgenetics.org

BlueSky: @plos.bsky.social

----------------------------------------------------

Comments from the reviewers (if applicable):

----------------------------------------------------

**Data Deposition**

If you have submitted a Research Article or Front Matter that has associated data that are not suitable for deposition in a subject-specific public repository (such as GenBank or ArrayExpress), one way to make that data available is to deposit it in the Dryad Digital Repository. As you may recall, we ask all authors to agree to make data available; this is one way to achieve that. A full list of recommended repositories can be found on our website.

The following link will take you to the Dryad record for your article, so you won't have to re‐enter its bibliographic information, and can upload your files directly:

http://datadryad.org/submit?journalID=pgenetics&manu=PGENETICS-D-26-00598R1

More information about depositing data in Dryad is available at http://www.datadryad.org/depositing. If you experience any difficulties in submitting your data, please contact help@datadryad.org for support.

Additionally, please be aware that our data availability policy requires that all numerical data underlying display items are included with the submission, and you will need to provide this before we can formally accept your manuscript, if not already present.

----------------------------------------------------

**Press Queries**

If you or your institution will be preparing press materials for this manuscript, or if you need to know your paper's publication date for media purposes, please inform the journal staff as soon as possible so that your submission can be scheduled accordingly. Your manuscript will remain under a strict press embargo until the publication date and time. This means an early version of your manuscript will not be published ahead of your final version. PLOS Genetics may also choose to issue a press release for your article. If there's anything the journal should know or you'd like more information, please get in touch via plosgenetics@plos.org.

# Editor report: Acceptance letter

**DOI:** 10.1371/journal.pgen.1012340.r005

**Authors:** Paula Cohen, Michael Metzger

PGENETICS-D-26-00598R1

Revisiting the impact of synthetic ORF sequences on engineered LINE-1 retrotransposition

Dear Dr Richardson,

We are pleased to inform you that your manuscript entitled "Revisiting the impact of synthetic ORF sequences on engineered LINE-1 retrotransposition" has been formally accepted for publication in PLOS Genetics! Your manuscript is now with our production department and you will be notified of the publication date in due course.

The corresponding author will soon be receiving a typeset proof for review, to ensure errors have not been introduced during production. Please review the PDF proof of your manuscript carefully, as this is the last chance to correct any errors. Please note that major changes, or those which affect the scientific understanding of the work, will likely cause delays to the publication date of your manuscript.

Soon after your final files are uploaded, unless you have opted out or your manuscript is a front-matter piece, the early version of your manuscript will be published online. The date of the early version will be your article's publication date. The final article will be published to the same URL, and all versions of the paper will be accessible to readers.

For Research Articles, you will receive an invoice from PLOS for your publication fee after your manuscript has reached the completed accept phase. If you receive an email requesting payment before acceptance or for any other service, this may be a phishing scheme. Learn how to identify phishing emails and protect your accounts at https://explore.plos.org/phishing.

Thank you again for supporting PLOS Genetics and open-access publishing. We are looking forward to publishing your work!

With kind regards,

Anitha Samidurai

PLOS Genetics

On behalf of:

The PLOS Genetics Team

Carlyle House, Carlyle Road, Cambridge CB4 3DN | United Kingdom

plosgenetics@plos.org | +44 (0) 1223-442823

plosgenetics.org | Twitter: @PLOSGenetics
